# Environmental and Biogeographic Drivers behind Alpine Plant Thermal Tolerance and Genetic Variation

**DOI:** 10.3390/plants13091271

**Published:** 2024-05-04

**Authors:** Lisa M. Danzey, Verónica F. Briceño, Alicia M. Cook, Adrienne B. Nicotra, Gwendolyn Peyre, Maurizio Rossetto, Jia-Yee S. Yap, Andrea Leigh

**Affiliations:** 1 School of Life Sciences, Faculty of Science, University of Technology Sydney, Broadway, NSW 2007, Australia; alicia.cook@uts.edu.au; 2 Research School of Biology, Australian National University, Canberra, ACT 2601, Australia; vbricenor@gmail.com (V.F.B.); adrienne.nicotra@anu.edu.au (A.B.N.); 3 Department of Civil and Environmental Engineering, University of the Andes, Bogota 111711, Colombia; gf.peyre@uniandes.edu.co; 4 Research Centre for Ecosystem Resilience, Australian Institute of Botanical Science, Royal Botanic Gardens Sydney, Sydney, NSW 2000, Australia; maurizio.rossetto@botanicgardens.nsw.gov.au (M.R.); samantha.yap@botanicgardens.nsw.gov.au (J.-Y.S.Y.); 5 Queensland Alliance of Agriculture and Food Innovation, University of Queensland, Brisbane, QLD 4072, Australia

**Keywords:** evolutionary ecology, heat tolerance, cold tolerance, landscape genetics, species distribution models, last glacial maximum

## Abstract

In alpine ecosystems, elevation broadly functions as a steep thermal gradient, with plant communities exposed to regular fluctuations in hot and cold temperatures. These conditions lead to selective filtering, potentially contributing to species-level variation in thermal tolerance and population-level genetic divergence. Few studies have explored the breadth of alpine plant thermal tolerances across a thermal gradient or the underlying genetic variation thereof. We measured photosystem heat (T_crit-hot_) and cold (T_crit-cold_) thresholds of ten Australian alpine species across elevation gradients and characterised their neutral genetic variation. To reveal the biogeographical drivers of present-day genetic signatures, we also reconstructed temporal changes in habitat suitability across potential distributional ranges. We found intraspecific variation in thermal thresholds, but this was not associated with elevation, nor underpinned by genetic differentiation on a local scale. Instead, regional population differentiation and considerable homozygosity within populations may, in part, be driven by distributional contractions, long-term persistence, and migrations following habitat suitability. Our habitat suitability models suggest that cool-climate-distributed alpine plants may be threatened by a warming climate. Yet, the observed wide thermal tolerances did not reflect this vulnerability. Conservation efforts should seek to understand variations in species-level thermal tolerance across alpine microclimates.

## 1. Introduction

Alongside water, temperature is arguably the most important factor influencing plant biological processes, distributions, and adaptation [1,2,3]. In alpine ecosystems, the thermal gradient associated with elevation disproportionately exposes plants to fluctuations in extreme temperatures [4]. Whilst very low temperatures are often considered synonymous with high elevations, small-statured plants with cold-acclimated leaves can also be exposed to damaging heat on treeless, sheltered slopes even when ambient temperatures are only moderately high [5]. As snowmelt timing varies across elevations, plants are vulnerable to unpredictable frosts during the growing season when not protected by an insulating layer of snow [6]. Furthermore, different topographies among global alpine systems could mean that the thermal effects of elevation may be less pronounced in low-relief alpine landscapes, such as the Australian Alpine Bioregion. Under climate change, the extent and frequency of temperature extremes are expected to increase [7], with high-elevation systems warming faster than other ecosystems [8,9]. Furthermore, warming temperatures have already led to a reduction in snow cover, depth [10,11], and earlier and more variable springtime snowmelt [12,13], conditions that can amplify frost damage to plants [14,15]. As such, plants inhabiting alpine ecosystems must cope with and continue to adapt to both hot and cold extremes with the severity and timing of such events broadly varying across elevation gradients.

Thermal tolerance is not a fixed trait [16] but rather is thought to be closely related to the dynamic temperatures that plants experience in their environment [14,17]. Notwithstanding more local influences on environmental temperature, the strong elevation thermal gradient in alpine environments may contribute to variation in thermal tolerance. The freezing tolerance of alpine plants has been found to increase with elevation (Austrian Alps; [6]; Chilean Andes; [18]). These studies have focused on high altitudes (between 2000 and 3400 m a.s.l), and it is relatively unknown whether expectations will be upheld in low-elevation alpine systems, which can represent a smaller gradient. In terms of heat stress, the plant heat tolerance of different species decreases with elevation in tropical montane environments (2 m to 750 m a.s.l., Panama; [19]) and across diverse vegetation types (192 m to 2880 m a.s.l., Colombia; [20]). Yet, to our knowledge, it is not known whether this relationship holds for species within alpine systems, where extremely high temperatures are less common. Moreover, the concurrent heat and cold tolerance of alpine plants across an elevation gradient has not been explored. Considering the large diurnal fluctuations in alpine temperatures that lead to both heat and cold stress in plants, the complete spectrum of upper and lower thermal limits, i.e., thermal tolerance breadth (TTB), would provide a more comprehensive understanding of thermal tolerance under highly variable and extreme conditions [21].

Plants distributed along strong environmental gradients, such as elevation, are subject to selective pressures and restricted gene flow among diverse habitats [22,23]. As such, environmental selection that culminates in genetic and phenotypic variation can lead to the local adaptation of populations. Whilst inferences of adaptation cannot be drawn from in situ studies alone, we can begin to unravel the genetic variability underpinning mechanistic traits by analysing neutral processes governed by gene flow. Neutral genetic variation is often evident across the geographic distribution of species, facilitated by isolation-by-distance (IBD) mechanisms that have long been described as the baseline pattern in landscape genetics [24]. However, environmental heterogeneity can influence migration between populations and lead to genetic variation across much finer scales [25,26]. Patterns in genetic variation across elevation gradients have been explored in montane regions (between 990 and 1540 m a.s.l, southern Spain; [25]); yet, alpine systems, where microclimates are generally more heterogenous [27], remain relatively understudied (but see [28]). Furthermore, it is unclear to what extent patterns in plant thermal tolerance across elevation are associated with underlying neutral genetic signals [29]. Due to strong environmental filtering across relatively fine scales, alpine plants distributed along elevation gradients offer an ideal system to study genetic and phenotypic responses to extreme temperature shifts.

Whilst the elevation range of alpine species is strongly driven by selective pressures across that range, such as temperature, the natural distribution of species on a geographic scale is also determined by historical processes. Globally, alpine biodiversity has been shaped by historical glacial and interglacial cycles throughout the Pleistocene [30,31,32]. During glacial oscillations, alpine species are generally expected to conform to either of two contrasting biogeographical patterns: (i) distributional expansion during glacial periods and contractions to higher elevations during interglacial periods or (ii) distributional reduction and fragmentation at the height of glaciation and expansion into newly habitable areas following glacial retreat [33]. The extent to which either of these climate-related distributional shifts plays out will vary depending on latitude, physical landscape, and species-specific ecological demands and dispersal potential [34,35,36]. These historical settings often have a stronger influence on population-level genetic composition than current selection pressures or gene flow [37], such that temporal changes in species distributions may leave discernible genetic patterns in extant populations [38]. Species distribution models (SDMs) can be used to corroborate landscape-level genetic patterns by reconstructing temporal changes in potential distributions, based on habitat suitability, under current and paleoclimatic conditions. Whilst genetic studies on Australian alpine biota begin to reveal observable genetic patterns from a paleobiogeographical perspective [39,40], to our knowledge, no studies in the Australian Alps have used SDMs to test these inferences. Understanding the historical processes that have shaped distributions and genetic architecture may uncover signals of plant responses to climatic shifts [41].

We examined broad patterns in thermal tolerance thresholds and neutral genetic variation using elevation as a proxy for steep thermal gradients in alpine landscapes. Specifically, we aimed to (1) determine whether elevation explains variation in photosystem cold and heat thresholds and the thermal tolerance breadth (TTB) of ten Australian alpine plant species; (2) assess the extent to which population-level genetic variation exists across elevation for all study species or across distributions for a subset of seven wider-ranging species, and whether associative patterns between thermal thresholds and underlying genetics can be drawn; and (3) investigate how historical processes may have shaped present-day genetic patterns of three focal species by modelling potential distributions under current and paleoclimate conditions. The study species selected represent dominant alpine plant life forms that are subject to air temperature decoupling. Considering aim 1, we expected to confirm one of two contrasting hypotheses: (i) aligning with existing research, cold thresholds would increase with increasing elevation and heat thresholds would decrease or (ii) contrasting current studies, thermal thresholds would be largely unrelated to elevation due to narrow elevation ranges characteristic of the Australian Alps and the overwhelming influence of heterogenous local environment. Under either hypothesis, thermal tolerance breadth was predicted to remain relatively consistent across the gradient, driven by either opposing elevational trends in heat and cold thresholds or by thermal thresholds being unrelated to elevation. Due to selective filtering along a strong environmental gradient and within a heterogenous alpine landscape, we hypothesised that limits to gene flow would be prevalent, resulting in genetic differentiation across elevation and local spatial gradients (aim 2). If we found thermal tolerance thresholds to vary with elevation, genetic divergence across the same gradient would suggest a genetic underpinning for tolerance variation. As alpine species are currently distributed across cool climates, we hypothesised that conditions of the last glacial maximum (LGM ~21,000 years ago) would facilitate a greater extent of suitable habitat for focal species (aim 3). Whilst expansion during glacial periods rather than contraction may counter some previous studies, our hypothesis considers the Australian context where alpine regions were subject to less glacial coverage during the Pleistocene than global alpine systems. If genetic variation was found to be largely unrelated to selective pressures across elevation and within the local environment, reconstructing temporal distributional dynamics could further unravel broad-scale and contrasting genetic patterns of three focal species.

## 2. Results

### 2.1. Thermal Tolerance Thresholds across Elevation and Species

**Figure 1:**
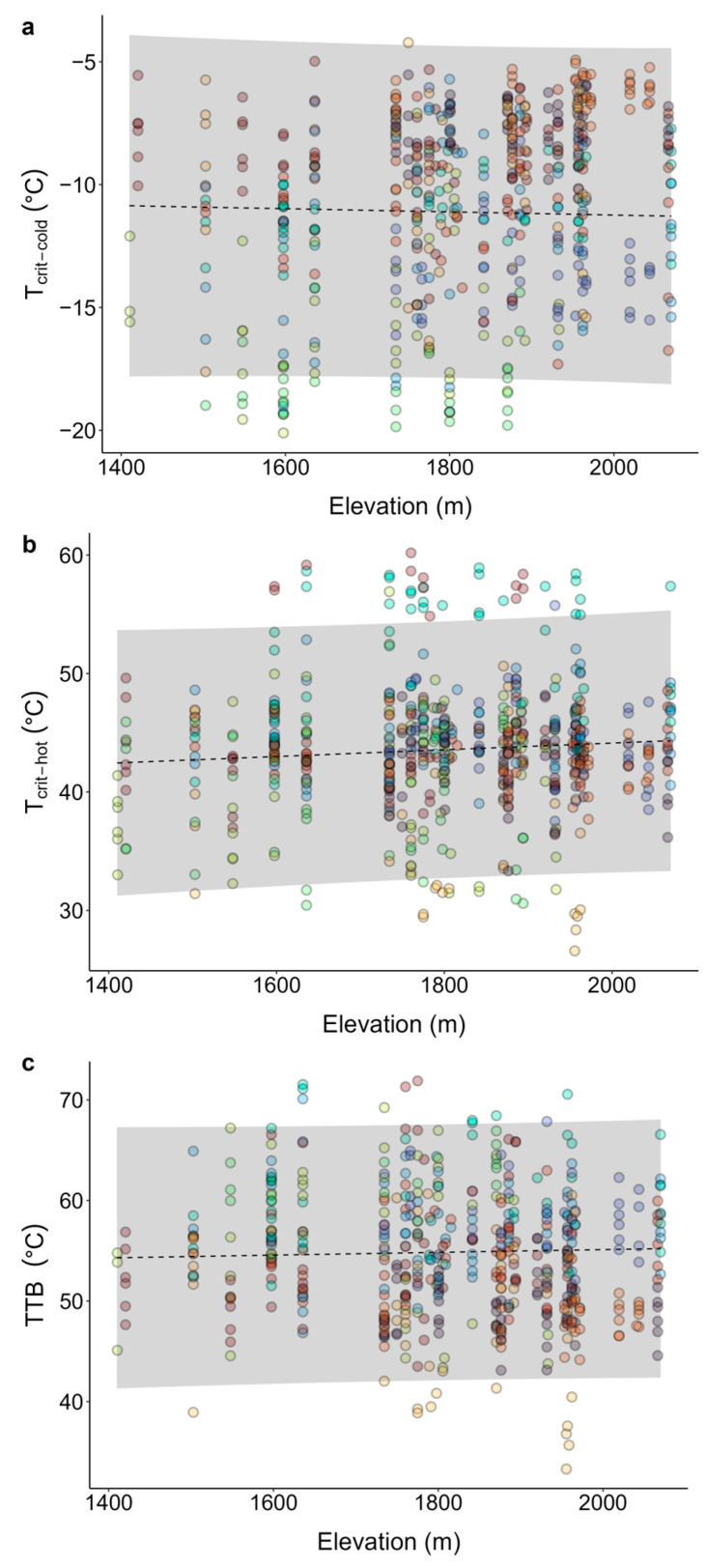
Thermal tolerance thresholds for ten alpine species along elevation gradients: (**a**) critical cold thresholds (T_crit-cold_), (**b**) critical heat thresholds (T_crit-hot_), and (**c**) thermal tolerance breadth (TTB). Circles represent observed thermal tolerance thresholds for individual leaf samples with colours representing different species. The regression lines and confidence intervals are the results of the linear mixed models accounting for species as a random factor (see Table 1). Additionally, the linear mixed model for T_crit-cold_ accounts for date as a random factor, and the model for T_crit-hot_ accounts for site as a random factor. Dashed model regression lines indicate that these are all nonsignificant relationships and shaded ribbons represent the 95% confidence intervals.

During the physiological experimental period, both the mean minimum and mean maximum air temperature significantly decreased along the elevation gradient (three consecutive days of concurrent recording across 13 temperature logging stations; Figure S1). Thermal tolerance thresholds were not significantly associated with elevation gradients (Table 1, Figure 1). However, there was considerable intraspecific and interspecific variation in thermal tolerance thresholds at a given elevation point for all parameters (see spread of datapoints in Figure 1). For all species across sites, cold thresholds (T_crit-cold_) ranged between −20.1 °C and −4.2 °C (Figure 1a), heat thresholds (T_crit-hot_) ranged between 26.7 °C and 60.2 °C (Figure 1b), and thermal tolerance breadth (TTB) ranged between 33.3 °C and 71.9 °C (Figure 1c). The proportion of variation in T_crit-cold_, T_crit-hot_, and TTB explained by combined fixed and random factors was 69% (elevation, species, and date), 36% (elevation, species, and site), and 45% (elevation and species), respectively, whilst a much smaller proportion of variation was explained by elevation alone (0.1%, 0.6%, and 0.1%, respectively; Table 1). The contribution of random effects provides evidence that factors other than elevation contribute strongly to variation in thermal thresholds. Because of the clear influence of species on variation in all thermal thresholds, irrespective of elevation, we further investigated the relationship between species and thermal thresholds independent of elevation. Species was a significant determinant of thermal thresholds (Table 2).

### 2.2. Patterns of Genetic Variation

Following SNP quality filtering, we performed analyses with 26,051 SNPs for *Aciphylla glacialis*, 11,645 SNPs for *Astelia alpina*, 23,439 SNPs for *Psychrophila introloba*, 9867 SNPs for *Richea continentis*, 8763 SNPs for *Epacris paludosa*, 19,231 SNPs for *Grevillea australis*, 6446 SNPs for *Prostanthera cuneata*, 13,575 SNPs for *Hovea montana,* 14,314 SNPs for *Oxylobium ellipticum*, and 5651 SNPs for *Tasmannia xerophila*.

**Figure 2:**
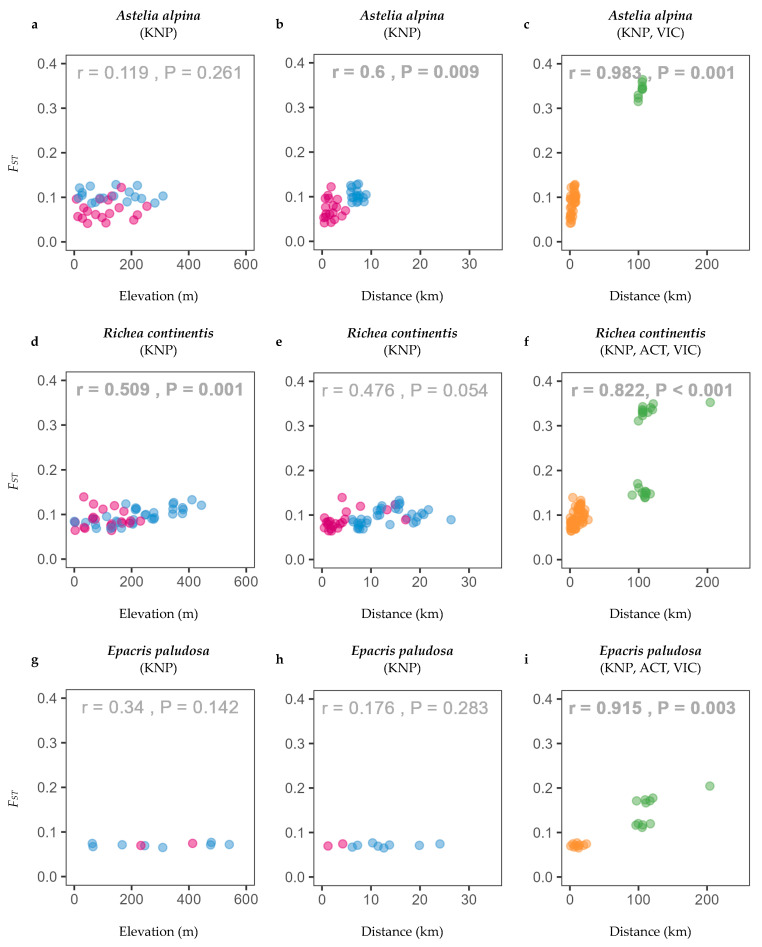
Isolation-by-distance plots comparing genetic differentiation (*F_ST_*) of alpine plant species across (**a**,**d**,**g**) elevation and (**b**,**e**,**h**) geographic distance in Kosciuszko National Park (KNP) and (**c**,**f**,**i**) across south-eastern Australia. Three example species are shown here: (**a**–**c**) *Astelia alpina,* (**d**–**f**) *Richea continentis*, and (**g**–**i**) *Epacris paludosa* (refer to Figure S2 for the results of all study species). For the plots in the left and middle panels, the coloured symbols represent within- and between-transect pairwise comparisons of sites (pink for within transects and blue for between transects). The study transects are Charlotte Pass, Perisher, and Thredbo. For the plots in the right panel, the coloured symbols represent within- and between-region pairwise comparisons of sites (orange for within regions and green for between regions). The study regions are Namadgi, ACT, Kosciuszko National Park, NSW, and Alpine National Park, VIC.

Generally, within the local range of Kosciuszko National Park, pairwise F_ST_ between populations remained relatively low (*F_ST_* < 0.2). Contrary to expectations, *F_ST_* was largely not correlated with elevation for study species across the investigated gradients, with the exception of *R. continentis* (r = 0.509, *p* = 0.001; Figure 2, Figure S2). Genetic differentiation significantly increased with distance for only three species within the local range (*A. alpina*, *P. introloba*, and *H. montana;* Figure 2b, Figure S2). There was between-transect genetic differentiation observed, yet the isolation-by-distance (IBD) pattern was relatively continuous within the local range such that admixture was likely occurring among transects. Across the wider geographic range of south-eastern Australia, *F_ST_* was significantly related to distance for all sampled species (seven species from two or three regions: NSW plus ACT and/or Victoria; Figure 2c,f,i, Figure S2). Although between-population pairwise *F_ST_* and geographic distance were significantly correlated across this range, there was clear within-region clustering of populations such that populations within regions were more genetically similar than among regions.

**Figure 3:**
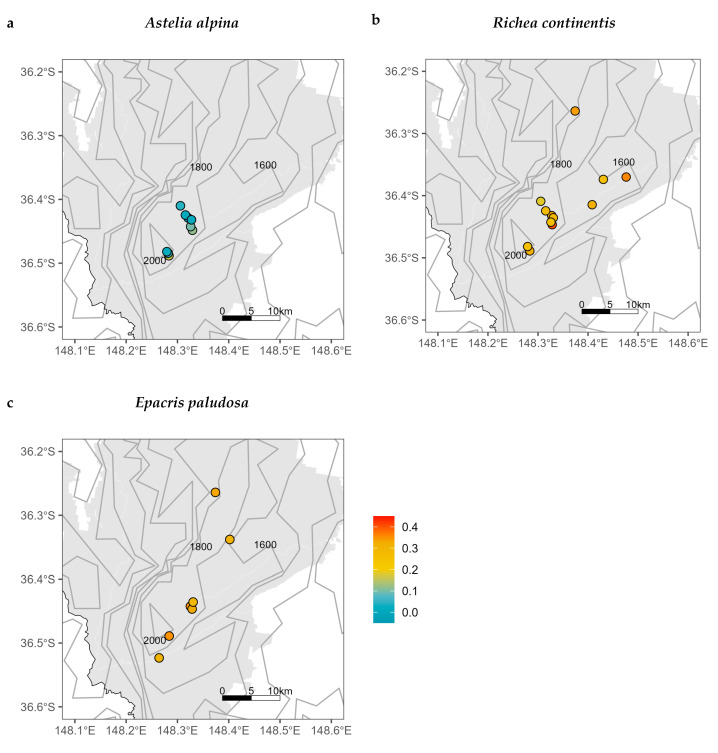
Within-population inbreeding (*F_is_*) of alpine plant species within Kosciuszko National Park, NSW. Three example species are shown here: (**a**) *Astelia alpina,* (**b**) *Richea continentis*, and (**c**) *Epacris paludosa*. Colours of circles indicate increasing *F_is_*. Figure S4 includes *F_is_* within Kosciuszko National Park as well as across south-eastern Australia for all ten species. The grey area represents the boundary of Kosciuszko National Park. The grey contour lines represent elevation with intervals of 200 m.

For all study species, populations largely exhibited inbreeding (*F_is_* > 0), with the exception of two populations of *P. cuneata* (Snowy River low: *F_is_* −0.015 and Munyang River: *F_is_* −0.005; Table S7). Intraspecific *F_is_* values were similar for populations across both local and regional spatial scales (Figure 3; Figure S4). Where within-species differences did exist, genetic diversity was inconsistently correlated with environment (latitude, longitude, and elevation) for four species within Kosciuszko National Park (*A. alpina, P. introloba, H. montana*, and *O. ellipticum*) and two species across the wider geographic range (*O. ellipticum* and *T. xerophila*; see Table S8 for species’ *p*-values and *rho*). There was clear interspecific variation in genetic diversity, where some species had higher ranges of *F_is_* and, as such, lower genetic diversity (e.g., *P. introloba*, *R. continentis*, *E. paludosa*, *G. australia*, and *O. ellipticum* average *F_is_* > 0.2; Table S7). Other species had lower ranges of *F_is_*, indicating more within-population genetic diversity (e.g., *A. alpina* and *P. cuneata* average *F_is_* < 0.1; Table S7).

Across elevation gradients, sites with paired thermal tolerance and genetic analyses were assessed for associative patterns. Considering population-level physiological traits, thermal thresholds (T_crit-cold_, T_crit-hot_, and TTB averaged per population) were unrelated to genetic distance (*F_st_*; Figure S3) and largely unrelated to diversity (Table S9). Genetic diversity significantly decreased with increasing thermal tolerance breadth for one species; *O. ellipticum* (*H_o_*: *p* = 0.042; *rho* = −0.829 and *F_is_*: *p* < 0.001; *rho* = 1.00). Whilst strong relationships between genetic metrics and thermal thresholds were not found, these results should be interpreted with caution due to low sample size when relying on paired population-level datasets (*n* = 4–8). Furthermore, it is important to note that averages of thermal thresholds per population may not adequately capture the considerable within-population variation in thresholds.

### 2.3. Temporal Changes in Habitat Suitability

To explore evolutionary drivers of contrasting genetic patterns among alpine species, we modelled habitat suitability under current and paleoclimate conditions for three focal species. Overall, the performance of the SDMs was good (average TSS > 0.6; Table S10). For all focal species, the RF algorithm performed best (ROC: 0.963 and TSS: 825), with 84–100% of algorithm runs kept for ensemble models. From the set of bioclimatic variables used in individual SDMs, the ensemble models weighted the variables based on relative importance for the potential distribution of each species. The bioclimatic predictors that were considered most ecologically relevant (variable importance > 50%) in forecasting potential distributions were a mix of temperature and precipitation-related factors. Both precipitation (bio14 = 29%) and temperature variables (bio9 = 45%) were important for distributions of *E. paludosa*, which possesses a wide longitudinal and latitudinal range, whereas temperature was the primary predictor of altitudinally limited distributions of *A. alpina* (bio8 = 23% and bio9 = 35%) and *R. continentis* (bio1 = 77%).

**Figure 4:**
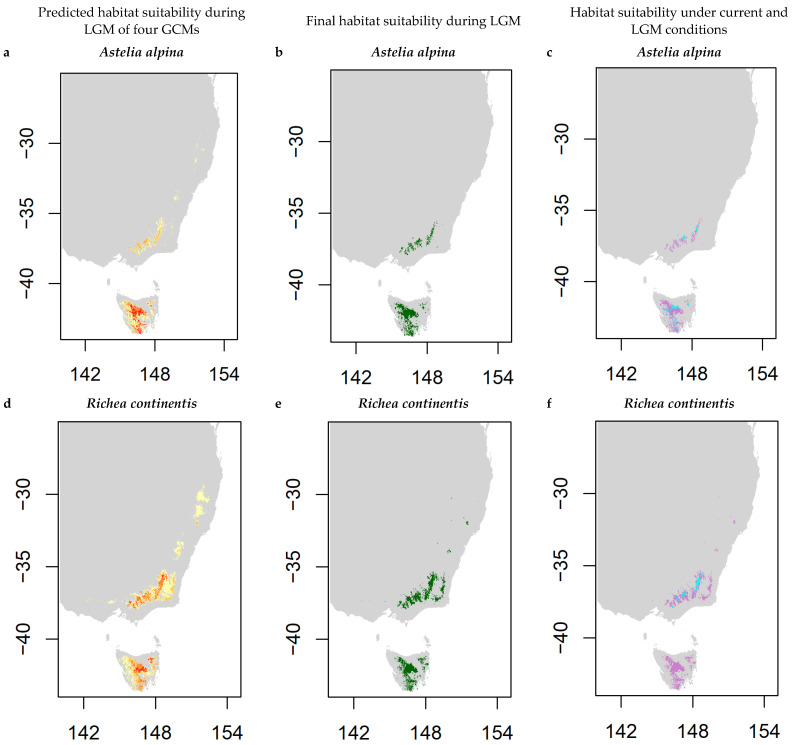

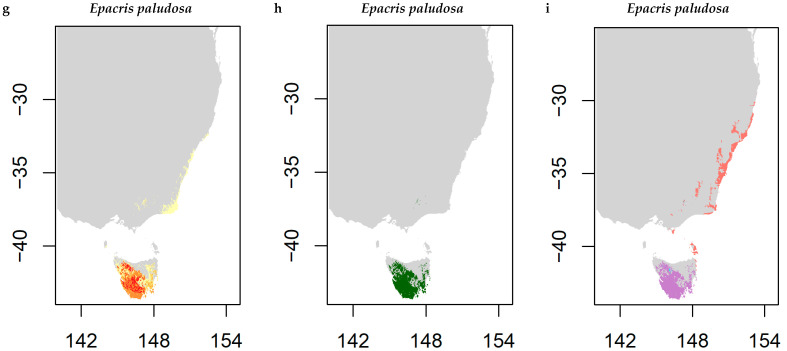
Modelled potential distributions, depicted as suitable habitat, during the last glacial maximum (LGM; approx. 21,000 y.a.) of three focal species, (**a**,**b**) *Astelia alpina*, (**d**,**e**) *Richea continentis*, and (**g**,**h**) *Epacris paludosa* and (**c**,**f**,**i**) their respective temporally stable habitat where current and LGM distributions overlap. For each species, (**a**,**d**,**g**) the predicted suitable habitat during the LGM across four global climate models (GCMs) are shown where colours represent the agreement of suitable habitat among GCMs: yellow (potential distribution predicted by one GCM scenario) to red (potential distribution predicted by four GCM scenarios). The final LGM potential distributions were decided where 50% of models agreed (**b**,**e**,**h**). Temporally stable habitats where species are predicted to exist currently and during the LGM are shown in blue, areas where species only exist currently are shown in pink, and areas where species only existed in the LGM are shown in purple (**c**,**f**,**i**).

By overlaying predictions of four global climate models (GCMs) and retaining areas where 50% of GCMs agreed on habitat suitability (Figure 4a,d,g), the suitable habitat during the last glacial maximum was 30,864 km^2^ for *Astelia alpina*, 50,318 km^2^ for *Richea continentis*, and 54,679 km^2^ for *Epacris paludosa* (Figure 4b,e,h). For the three focal species, SDMs predicted a loss in suitable habitat over time with contraction since the LGM, with values of 80% for *A. alpina*, 90% for *R. continentis*, and 26% for *Epacris paludosa* (Figure 4c,f,i). During the LGM, both *A. alpina* and *R. continentis* exhibited broader potential distributions, as described here by habitat suitability, across south-east Australia and Tasmania, with some fragmentation across this range, whilst *E. paludosa* was mostly distributed in Tasmania during this period. For *A. alpina* and *R. continentis*, suitable habitat decreased over time, but the location of the continuous distributional area of each species did not shift strongly, except that *R. continentis* did not remain in Tasmania. Both species exhibit relatively comparable land coverage where the habitat suitability of the LGM and current potential distributions overlap, hereafter referred to as temporally stable habitat. *Astelia alpina* was predicted to have 5411 km^2^ of temporally stable habitat and *R. continentis* was predicted to have 4607 km^2^ of temporally stable habitat (Figure 4c,f). On mainland Australia, *Richea continentis* has a larger area of temporally stable habitat than *A. alpina*, which is also distributed in Tasmania. On the other hand, the potential distributional area of *E. paludosa* largely shifted between the LGM and now. During the LGM, models predicted that *E. paludosa* was extant in Tasmania and small areas of mainland Australia near the Victorian Alps. The current distribution of *E. paludosa* is fragmented across the south-east of the mainland, Flinders Island (between the mainland and Tasmania), and in small areas in northern parts of Tasmania. Due to the northward shift in the distribution of *E. paludosa*, there are only small areas (414 km^2^) of temporally stable habitat in northern Tasmania and the Victorian Alps (Figure 4i).

## 3. Discussion

In high-altitude alpine systems, plants’ freezing tolerance increases with elevation [6,18], but there is no evidence for a corresponding negative relationship for alpine plant heat tolerance (but see [20]). For the low-altitude Australian Alps, we addressed the assumption that elevation thermal gradients drive thermal tolerance with two contrasting hypotheses: Cold and heat tolerance thresholds would either (i) follow the currently published patterns of a positive elevational trend for cold thresholds and a negative elevational trend for heat thresholds or (ii) would not vary with elevation, and that either outcome would result in no change in thermal tolerance breadth. Our second expectation was supported: Neither cold nor heat thresholds, or TTB, were related to elevation for our study species. Instead, the marked species-level variation in all thermal thresholds suggests that thermal tolerance is more complex than can be predicted by broad-scale environmental influences alone. Contrasting our hypothesis that environmental heterogeneity would lead to local genetic divergence, but corroborating mechanistic findings, we found little conclusive evidence for strong adaptive barriers associated with elevation, as inferred by neutral genetic differentiation. Rather, population differentiation, although shallow, appears to be explained by geographic distance, particularly on a regional scale. As we hypothesised, habitat availability for three focal species was projected to be wider under the cooler, drier climate of the last glacial maximum (LGM). Yet, besides the contraction of suitable habitat following the LGM, the forecasted distribution dynamics were not identical for all three focal species, with implications for current broad-scale genetic patterns. Below, we explore the potential drivers of alpine plant thermal thresholds and population-level genetic patterns considering historical changes in suitable habitats.

### 3.1. Drivers of Species-Level Thermal Tolerance Variation

One reason that our results did not reflect expected elevational trends in cold thresholds could be an overriding influence of the local environment, such as differences among microenvironments. Alpine landscapes consist of a mosaic of heterogenous microenvironments that are largely governed by snow cover and melt regimes linked to complex combinations of wind, topography, and aspect, all interacting with the effects of elevation [27]. When plants in early snowmelt sites are exposed to more intense and frequent freezing events than plants at late snowmelt sites, these conditions are known to increase cold hardening, or acclimation, in the following season [42,43,44]. Intraspecific variation in cold thresholds at a given elevation suggests that microclimatic effects are a strong driver of heterogenous physiological response across very fine scales, which may be obscured by broad-scale elevation.

The heat tolerance of plant photosystems has been found to decrease with elevation when lower elevations include tropical forests, where potentially damaging high temperatures are more frequent [19,20]. In alpine systems, high air temperatures capable of impairing photosystems are far less likely (maximum summer temperature of 29.8 °C over the last 30 years at Perisher Valley [45]); yet, we found that across all ten species, relatively high mean heat thresholds (>40 °C) were maintained. In support of our findings, high heat tolerance has been recorded for plants in the European [5,46] and Australian Alps [47]. As observed for cold thresholds, there remained considerable variation within species and sites. During late spring and early summer, alpine plants, particularly at high elevations, can experience large fluctuations in temperature (up to 20 °C within a single day during this study; Figure S1), such that plants need to cope with extremely low and relatively high temperatures over a single day [48]. Where plants are exposed to compounding environmental pressures, common cellular responses are known to increase protection against multiple stresses [49]. Note that, in our study, although *average* air temperatures varied significantly with elevation, the temperature *range* remained relatively constant (Figure S1). We would expect large diurnal temperature fluctuations to select for the wide thermal tolerance breadth that was maintained across the elevational range, which was indeed what we found.

A compelling finding of this study was that irrespective of elevation, species consistently explained the most variation in thermal thresholds. Corresponding with our findings, neither elevation nor snowmelt gradients were found to significantly influence the cold tolerance of alpine shrubs, which varied more clearly with species [44]. The observed variation in thermal thresholds among our ten species is likely driven, at least in part, by distinct thermal niches. The breadth of an ecological niche is said to predict geographic ranges due to selective filtering across the landscape [50]. Similarly, plant populations distributed across a strong environmental gradient are subject to selection on a local scale [51] such that the elevation range may similarly be determined by thermal niche. In this study, species had varying breadths of both geographic and elevation ranges. Irrespective of the span of elevation, the position of species-specific ranges along elevation gradients differed between low-elevation-distributed species (e.g., *Tasmannia xerophila;* mean TTB of 54.8 ± 0.7 °C) and species restricted to high elevations (e.g., *Psychrophila introloba;* mean TTB of 48.2 ± 0.8 °C). Thermal niches are most likely more nuanced than can be explained by simplistic range limits [52], with microhabitats and intrinsic species traits, such as growth form, at play within these ranges [14,44]. As such, interspecific differences in thermal thresholds may reflect species’ fundamental thermal niche associated with microhabitats within their distinct elevation range.

### 3.2. Population Genetic Patterns Underlying Thermal Tolerance Variation across Elevation and Geographic Gradients

Both intraspecific and interspecific variations in thermal thresholds reflect differing extents of within-generation acclimation and intergenerational adaptation, most likely a combination of both. In our study, we explored neutral patterns in genetic structuring among alpine plant populations across elevations that may infer the presence of local adaptation to thermal conditions. Within the confined range of Kosciuszko National Park, genetic differentiation (as measured by *F_ST_*) for all species remained low, and where significant differentiation among populations did exist, it was largely unrelated to elevation. Narrow elevation gradients of Australian alpine systems are thought to facilitate gene flow among populations that might overwhelm selection for local adaptation [53,54]. Therefore, the presence of intraspecific variation in thermal thresholds in the absence of strong genetic differentiation supports the idea that acclimation drives alpine plant response to microclimatic thermal extremes.

When examining differentiation across a wider geographic range (i.e., south-eastern Australia), genetic divergence between regions became more pronounced across all species. Population divergence and structuring at this scale are likely driven by temporal isolation and vicariance processes acting across the distribution of species. However, for our study species, overall genetic differentiation remained relatively shallow, even at a regional scale (*F_ST_* < 0.4). This shallow differentiation may be attributed to the continuing connectivity of populations through time or the rapid expansion of species. Interestingly, seemingly unrestricted gene flow among populations was contradicted by relatively high levels of within-population inbreeding (as measured by *F_is_*) at both local and regional scales. The influence of landscape on considerable homozygosity within populations is not readily apparent. Among different species, genetic diversity measures (here, allelic diversity) were inconsistently correlated with latitude, longitude, and elevation. Previous studies have identified post-glacial recolonisation as explaining latitudinal trends in genetic diversity [55,56] and to a lesser extent longitudinal trends [57]. It is likely that a combination of environmentally and geographically isolating factors over time have differentially influenced species’ genetic diversity, depending on distinct breeding systems, dispersal mechanisms, and genetic controls.

Finally, varied topography may have shaped the gene flow at a regional scale. Although the shallow genetic differentiation observed in our NSW alpine populations reflects the patterns observed in some global alpine systems (e.g., [58,59]), it differs from the deep divergence of mountain top plant populations found in the Victorian populations of Australian Alps [39]. It is possible that regional differences in the physical landscape are responsible for the distinct genetic architecture in Victorian and NSW alpine populations, as has been found in other alpine systems [22]. Low genetic differentiation and evidence of inbreeding may be explained by a history of glacial periods in high-elevation (>2000 m a.s.l) NSW alpine areas that were not present in Victoria’s relatively lower-elevation alpine regions [60]. The focused comparisons of genetic differentiation in plant populations across alpine regions of south-eastern Australia would help to uncover diverging genetic patterns, with associated implications for species conservation in differing regions.

### 3.3. Genetic Patterns in the Context of Temporal Changes in Habitat Suitability

Paleohistory in arctic and alpine regions has a strong influence on population-level genetic variation [37]. In light of the relatively low measures of genetic diversity (as indicated by inbreeding) of our alpine species, alongside unrestricted gene flow (as indicated by genetic differentiation), we were interested in understanding how broad-scale distributional dynamics may have left an imprint on present-day genetic factors. Specifically, reconstructing and comparing distributions between glacial (LGM) and interglacial periods (present day) could point to distributional dynamics that historically facilitated population inbreeding such as bottlenecks that temporally isolated populations.

The predicted habitat suitability was found to be much wider for the three focal species during the last glacial maximum, supporting our hypothesis. The cooler and drier conditions of the LGM likely facilitated the spread of alpine species to lower elevations [33]. Of the three focal species, the contraction of suitable habitat since the LGM was most evident for *A. alpina* and *R. continentis*, which had projected shifts to higher elevations. Despite both species following similar temporal dynamics in suitable habitats, they exhibited differing genetic patterns. *Astelia alpina* had relatively low levels of inbreeding (Figure 3a), potentially facilitated by population connectivity and persistence through time. By comparison, *R. continentis* showed higher levels of inbreeding (Figure 3b). One explanation for this discrepancy between the two species could be environmental and dispersal barriers during distributional expansion and contraction cycles [61] that were not entirely captured in the SDMs. This idea is supported by associations between genetic differentiation and landscape gradients for both species. For *A. alpina*, mostly restricted to high elevations, *F_ST_* was significantly related to distance locally (within Kosciuszko National Park; Figure 2b), suggesting that genetic structuring may be driven by the divergence of mountain top populations following range contractions to high elevations [39]. Conversely, *R. continentis*, distributed across wider elevation ranges, showed local genetic structuring across elevation rather than distance (Figure 2d), indicating the potential for present-day selective filtering leading to population differentiation across environmental gradients.

In contrast to these two species, the distribution of *E. paludosa* was projected to shift towards lower latitudes since the LGM, facilitating a recent migration of *E. paludosa* to expansive areas of mainland Australia from a southern source. The influence of recent migration on the genetic makeup of *E. paludosa* is underscored by comparatively low levels of genetic differentiation, both regionally and locally. As such, the gene flow that was unimpeded by landscape barriers likely aided the migration of *E. paludosa*, potentially culminating in reduced genetic diversity as the species migrated to new habitats [62]. The contrasting distributional shifts among focal species suggest that long-term persistence or recent migration may be the precursor of the differing genetic patterns observed among species today. With a focus on exploring paleodistribution, it was beyond the scope of this study to account for dispersal mechanisms in SDMs. However, it is crucial that research aiming to model temporal changes in active and realised distributions considers dispersal limitations across potential distributions [63,64]. These considerations are particularly important in alpine systems, where biogeographical barriers and steep environmental gradients often reduce the dispersal capacity of plants [65].

### 3.4. Considerations for Conservation of Alpine Landscapes under a Changing Climate

Our findings suggest that the common expectation that elevation thermal gradients drive alpine plant thermal tolerance is overly simplistic. Whilst our ten species varied widely in thermal tolerance responses, this variation was not associated with elevation. Corroborating mechanistic patterns, or lack thereof, genetic differentiation was not strongly associated with elevation. Habitat suitability for three focal study species appeared to be greater under LGM conditions, with distributions encompassing lower elevations, where temperatures were cooler than today. Yet, despite the projected cool-climate distributions, all study species showed considerably high heat thresholds. There are three potential explanations for this paradox. First, for plant species across a range of biomes, innate heat thresholds have been found to differ relatively little, supporting findings that high basal heat tolerance in plants is ubiquitous [66]. Second, plastic adjustments of the physiology of plants in a variable and extreme alpine climate could drive acclimation to different microclimates [5]. Finally, competition at lowland habitats may exclude alpine species, even though these species can cope with the warmer temperatures at lower elevations.

Based on our findings, we recommend that the management of alpine plants should steer focus away from elevation-driven responses, such as the assumption that species with wide elevation ranges are less at risk of being affected by the changing climate than high-elevation-restricted species. For example, the widely distributed species *R. continentis* had narrower TTB and lower genetic diversity than the high-elevation species *A. alpina.* Although our SDMs modelled historic range contraction for both species, the lower genetic diversity and local population structuring of *R. continentis* suggest that this species may be of greater concern with respect to adaptive capacity. Therefore, focus might be directed towards species-specific thermal limits and genetic makeup in alpine microclimates, particularly above the tree line. Whilst we were unable to conclusively link thermal thresholds with environmental drivers in this study, it is likely that the intraspecific variation in thresholds is related to fine-scale microclimates. The presence of thermal niches among different microhabitats may favour the persistence of different alpine plant communities [67] and aid in identifying potential refugia. Yet, there remains a limited understanding of how thermal tolerance variation is tied to environmental conditions on such a fine scale, a clear area for future research. It is important to note that shallow genetic differentiation alongside considerable phenotypic variation in thermal thresholds may actually infer robustness to variable climate via acclimatory responses [54]. Nonetheless, the prevalent homozygosity within alpine populations does signal the need for caution with respect to the adaptive capacity of species. Focused genetic studies should explicitly determine the role of acclimatory vs. adaptive plant responses to extreme and diurnally variable temperatures in alpine landscapes.

## 4. Materials and Methods

### 4.1. Field Study Area

**Figure 5:**
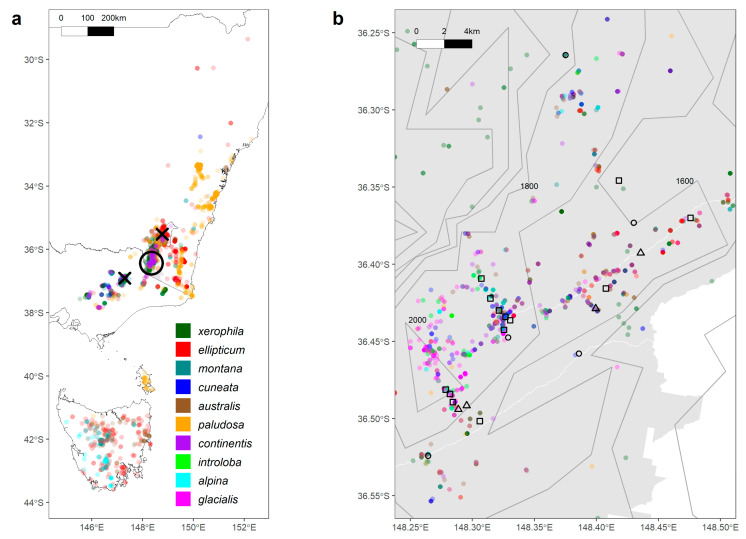
Maps showing the distributional and sampling range of ten alpine plant species across south-eastern Australia (**a**) and within Kosciuszko National Park (**b**). Refer to Table 3 for the full species list. Each coloured point represents cleaned floristic data sourced from Atlas of Living Australia [69], where different colours represent species. On the regional scale (**a**), the circle shows the approximate range of sampling across Kosciuszko National Park and crosses represent sites in Namadgi National Park, Australian Capital Territory (northern cross), and Alpine National Park, Victoria (southern cross). Within Kosciuszko National Park (**b**), symbols represent sample collection types: squares indicate sites where both genetic and physiological samples were collected, circles indicate sites where only genetic samples were collected, and triangles indicate where only physiological samples were collected. The grey area represents the boundary of Kosciuszko National Park (**b**), and the black borders on each map represent state boundaries for the Australian Capital Territory (ACT), New South Wales (NSW), and Victoria (VIC). Grey contour lines represent elevation with intervals of 200 m.

Field sampling was largely conducted in Kosciuszko National Park in New South Wales (NSW; Figure 5). Located in south-eastern Australia, Kosciuszko National Park has a mean annual temperature of 3–12 °C and a mean annual precipitation of 606–2344 mm [68]. We collected samples for both thermal tolerance and genetic analyses from sites distributed across three elevation gradients in Perisher Valley, Charlotte Pass, and Thredbo ski resorts (Table S1). Across each gradient, four to seven sites were selected that were separated by intervals of approximately 100 m in elevation. Collection sites were selected to represent the elevational range of each study species within Kosciuszko National Park, as far as possible (Table 3). As the natural elevational range of species varies, the set of species collected at each site often differed, but collections aimed for the overlap of at least three species at each site, and each species represented at a minimum of three sites of each gradient. Microsite logging stations measuring air temperature were established at key sites across the elevation gradients to confirm the existence of the elevation thermal gradient during the thermal tolerance field campaign. For wide-ranging species, genetic samples were collected from representative sites in Victoria (VIC) and the Australian Capital Territory (ACT) to corroborate the genetic patterns observed in Kosciuszko National Park (Figure 5a). The thermal tolerance field campaign was conducted early in the alpine growing season during December 2021. Genetic sampling was undertaken throughout 2021 during snow-free periods.

### 4.2. Species Selection

Ten alpine plant species were selected for thermal tolerance assays and genetic analyses, spanning a range of plant families and growth forms characteristic of Australian alpine vegetation communities (Table 3). The vegetation communities of the Australian Alps Bioregion are influenced by both elevation and precipitation with four distinct categories: alpine (above treeline, e.g., <1850 m a.s.l), subalpine (1400–1850 m a.s.l), montane (1100–1400 m a.s.l), and tablelands (<1100 m a.s.l) [70]. The species selected occurred above the tree line but differed in elevational range. The geographical boundaries of the study species varied from narrow-ranging species restricted to the Australian Alps Bioregion to wide-ranging species found across south-east Australia, including mainland states and territories of Victoria (VIC), New South Wales (NSW) and the Australian Capital Territory (ACT), and the island state of Tasmania. Three focal species from the full species set were selected for distribution modelling (see Section 4.5).

### 4.3. Field Sampling, Measurements, and Data Collection

#### 4.3.1. Microsite Logging Stations

Ambient air temperature was measured along three elevation gradients during the thermal tolerance field campaign to confirm the presence of elevational thermal gradients in situ. Between 4 and 6 sites per gradient were chosen for recording air temperatures, with a total of 13 microsite stations across the transects (Table S1). For each gradient, site selection for microsite logging stations included the highest and lowest elevation sites to encapsulate the entire extent of the elevational range relevant to the study. The deployment of stations across study sites was staged for logistical reasons, resulting in 6–10 days of continuous data for each station, with 3 overlapping days of temperature data for all sites.

At each site, microsite logging stations were installed to measure the air temperature at the approximate shrub canopy level (~5 to 100 cm above ground level) using fine-wire type-T thermocouples (36-gauge, Omega Engineering, Norwalk, CT, USA), connected to HOBO dataloggers (UX120-014M, HOBO^®^ Dataloggers Onset, Bourne, MA, USA). The thermocouple was attached to a stake or branch and shielded from direct sunlight with a small plastic white cup. Mean temperatures were recorded every 1 min of the temperatures that were measured every 5 s within a 1 min period. The daily mean minimum (T_min_) and daily mean maximum temperatures (T_max_) were taken as the lowest and highest of these 1 min means over 24 h. The daily mean minimum and mean maximum temperatures were considered representative of extreme temperatures that plants regularly experience over a given day.

#### 4.3.2. Thermal Tolerance Sampling and Assays

The measurement of photosystem thermal thresholds was conducted on field-collected leaf samples in a field laboratory at Charlotte Pass, NSW (−36.4357, 148.3333; elevation: 1785 m a.s.l) on the day of collection. For a given elevation gradient, assays were conducted over two consecutive days. At each site, leaves from six replicate plants per species were collected from individuals growing at least 5 m apart. Healthy, mature leaves were sampled from the outer, sun-exposed canopy. To prevent changes in leaf water status, leaves were placed into humid zip-lock plastic bags with moistened paper towels and kept at ambient temperature (~15 °C) in the dark until assays.

The photosynthetic machinery within the chloroplasts of leaves, particularly photosystem II (PSII), is susceptible to both heat [71,72] and cold stress [73,74]. Chlorophyll fluorometry can be used to determine photosynthetic thermal thresholds by measuring the functional response of PSII to temperature stress. The critical thermal thresholds of PSII (T_crit_) signify the onset of inactivation of PSII. T_crit_ is determined by measuring the temperature-dependent increase in chlorophyll fluorescence (T-F_0_) where the sudden increase in the emission of baseline fluorescence (F_0_) in response to a change in temperature determines T_crit_ [75]. Heat and cold tolerance thresholds of PSII were measured following the protocol outlined by Arnold et al. [76]. Briefly, a temperature assay involved exposing leaves or leaf sections to a heating or cooling ramp on a thermoelectrically controlled Peltier plate (CP-121HT; TE-Technology, Inc., Traverse City, MI, USA; 152 × 152 mm surface). Leaf samples were placed on an array on a white paper grid (5 × 9 cells) that was attached to the surface of the Peltier plate. A 40-gauge type-T thermocouple (Omega Engineering, Norwalk, CT, USA) was attached to the underside of each leaf to record leaf temperature every 10 s by a 16-channel datalogger (DataTaker DT85, Thermo Fisher Scientific, Waltham, MA, USA). Heavy double-glazed glass was placed on top of the leaf samples on the plate to maximise contact between samples, the attached thermocouples, and the surface of the Peltier plate.

A pulse amplitude-modulated (PAM) chlorophyll fluorescence imaging system (Maxi-Imaging-PAM; Heinz Walz GmbH, Effeltrich, Germany) was mounted 185 mm above the Peltier plate to measure fluorescence. A red perspex hood filtered light and a thick black fabric cover ensured that no actinic light reached the dark-adapted leaves during the experimental run. Prior to each experimental run, circular areas of interest (AOI) were selected for each of the 45 leaf samples using the ImagingWin software (PC software ImagingWinGigE2.56p). After leaves were dark-adapted for 30 min, a modulated measuring light was set to a continuous weak blue pulse (0.5 µmol photons m^−2^ s^−1^) at a low frequency (1 Hz) to determine minimal fluorescence (F_0_). Leaves were then exposed to a single saturating pulse (4000 µmol photons m^−2^ s^−1^) for 720 ms to determine the maximal fluorescence (F_M_) when the photosystem reaction centres were closed. The maximum quantum yield of PSII (F_V_/F_M_) was then calculated as (F_M_ − F_0_)/F_M_. F_V_/F_M_ is commonly used as a rapid method for measuring the health of photosynthetic tissue, with an F_V_/F_M_ below 0.2 representing critically damaged leaves and above 0.8 representing healthy, nonstressed leaves [77,78]. After obtaining pre-assay fluorescence parameters, the temperature ramp-up or ramp-down commenced.

A prescribed ramp setting was applied for the heating and cooling experimental runs to generate temperature-dependent chlorophyll fluorescence curves (T-F_0_). LabVIEW-based control software (VI LabVIEW2014; National Instruments, Austin, TX, USA) controlled the Peltier plate temperature ramping rate. For both hot and cold runs, the starting temperature for the experimental run was set to the ambient temperature of the field lab (10–15 °C). The ramp-up rate for heat T-F_0_ measurements was 30 °C per hour to reach a maximum temperature of 65 °C. The cold T-F_0_ measurements ramp-down rate was 15 °C per hour, reaching a minimum temperature of −20 °C. Critical thresholds were extracted by calculating the inflection point on each resulting T-F_0_ curve using break-point regression analysis in the *segmented* package [79] in R version 4.3.1 [80] and RStudio version 4.3.1 [81]. The thermal tolerance breadth (TTB) was determined as the difference between critical cold and heat thresholds (T_crit-cold_ and T_crit-hot_, respectively). The thermal tolerance threshold dataset was cleaned prior to statistical analysis by removing samples where the starting F_V_F_M_ was less than 0.6 and/or T-F_0_ curves that did not show a clear inflection point for T_crit_ to be extracted. The sample exclusion resulted in species and sites with unequal sample sizes.

#### 4.3.3. Genetic Sampling, Sequencing, and Analyses

The sampling strategy and field data collection for the genetic component followed protocols based on the *Restore & Renew* framework for plant ecological genomics research [82]. At each site, six to twelve individuals per species were collected, adopting a minimum spacing of 5 m between individuals. Where genetic samples were collected from the same populations as thermal tolerance samples, the datasets were not matched to the individual level. Fresh leaf tissue sampled from each individual was kept cool and dry prior to return to the lab where sample processing involved snap-freezing the sample at −80 °C and freeze-drying to enable the storage of material in silica gel until used for DNA extraction.

DNA extraction and the genotyping of samples were outsourced to Diversity Arrays Technology Pty Ltd. (DArT; Canberra, ACT, Australia), which applies a genotyping-by-sequencing platform called DArTseq [83]. This is a high-throughput approach that combines a genome complexity reduction method and next-generation sequencing (NGS) platforms described by Kilian et al. [84] and Cruz et al. [85]. DArTseq data contain a set of co-dominant single-nucleotide polymorphisms (SNPs) that are useful for understanding relationships among populations at the landscape level [86]. The NGS libraries were processed using proprietary DArT analytical pipelines that remove poor-quality sequences, call quality SNPs, and run a BLAST search of all loci to remove potential microbe contaminants.

After receiving the processed SNP datasets for each species, we performed further quality control and SNP filtering using a workflow developed by the *Restore & Renew* protocol [82] in R version 4.3.1 [80] and RStudio version 4.3.1 [81]. For each species dataset, filtering steps included the removal of SNP loci of poor quality, defined as a reproducibility average of less than 0.96 and genotypes missing in more than 20% of samples, and the removal of poor-quality samples that were missing data in a large proportion of loci (samples missing data in >20% of loci). From this subset of SNP data, SNPs were filtered to only include one SNP per locus to prevent the potential influence of linkage. For population-level analyses, minor allele frequency was set to 0.05.

### 4.4. Statistical Analysis of Field-Based Datasets

#### 4.4.1. Thermal Tolerance Thresholds across Elevation Gradients

First, to confirm the presence of the elevation thermal gradient during the physiological field campaign, linear mixed models with daily mean maximum temperature (T_max_) or daily mean minimum temperature (T_min_) as continuous response variables and elevation as the fixed continuous explanatory variable were computed (see Table S2 for model fitting). For temperature response models, the date of sampling (categorical, 3 levels) was included as the random effect to account for weather differences among sampling days. The additional random effects, transect (3 levels) and site (13 levels), and site nested within the transect were also considered to account for local effects of site aspect and slope and the spatial grouping of sites within the three elevation gradients, respectively. However, the random effects of site and transect led to singularity issues and did not improve models; thus, only the date of sampling was included.

Linear mixed models were used to determine the relationship between elevation and thermal tolerance thresholds (T_crit-cold_, T_crit-hot_, and TTB) with consideration of species-specific responses. Two families of models were examined. The first family had the fixed explanatory variable as elevation (continuous) and species as a random effect (categorical, 10 levels). The random effect of species was included in the models as thermal thresholds are known to vary intra- [87,88] and interspecifically [20,89], and species-level variation was of interest a priori (see Table S3 for model fitting). The T_crit-cold_ model included the date of sampling (categorical, 8 levels) as an additional random effect and the T_crit-hot_ model included site (categorical, 17 levels) as an additional random effect. The TTB model included only species as a random effect based on the best fit. For these models, both random intercepts and random slopes for elevation within each level of species were fitted. In the end, random slopes were excluded from models as the variance accounted for by species-specific slopes across elevation was effectively zero, leading to singularity issues for the models.

Given the importance of species in predicting thermal thresholds, the second family of models excluded elevation and had species as the sole fixed explanatory variable (categorical, 10 levels), investigating if species were the primary driver, irrespective of the environment (see Table S4 for model fitting). The T_crit-cold_ model included date as the random effect, and the T_crit-hot_ model included site as the random effect. The TTB model had species as the fixed effect and no random effects based on singular fits when including random effects. The mean and standard error for each species T_crit-cold_, T_crit-hot_, and TTB based on respective models were extracted from estimated marginal means’ post hoc analyses. As was applied for the elevation thermal gradient analyses, transect, site, date of collection, and site nested within the transect were initially included as random factors during model fitting for both model families, individually and in combination, where models would converge.

All linear mixed models were selected based on the lowest Akaike Information Criterion (AIC), significant differences among models, and the level of variance explained by random effects. Assumptions of normality and homoscedasticity for the final models were visually assessed with Q-Q plots and residual scatter plots, and the selected models satisfied the assumptions. All statistical analyses were conducted using R version 4.3.1 [80] and RStudio version 4.3.1 [81], with linear mixed models fit with the lme4 [90] package and post-hoc analyses performed using the emmeans [91] package. Figures were created in R with ggplot2 [92] and ggeffects [93] packages.

#### 4.4.2. Genetic Differentiation and Diversity across Sampling Ranges

We estimated patterns of gene flow across elevation and the wider distribution as between-site genetic differentiation based on pairwise *F_ST_* [94,95] with a 95% bootstrap confidence interval (999 replicates) in the R package SNPrelate [96]. The relationship between elevation and genetic distance and the magnitude of isolation by distance (IBD) was determined using a Mantel test with 10,000 random permutations in the R package vegan [97,98]. To test for associations between genetic differentiation and thermal tolerance, additional Mantel tests were performed using pairwise T_crit-cold_, T_crit-hot_, and TTB. As genetic and physiological datasets were not paired to the individual, we determined average thermal thresholds per population to use in genetic analyses.

Genetic diversity metrics, the expected (*H_e_*) and observed heterozygosity (*H_o_*), the inbreeding coefficient (*F_is_*), and allelic richness (*ar*) were estimated using the R package diveRsity [99]. Allelic richness was calculated using the built-in repeated random sampling technique (999 bootstrap replications) to correct for sites of different sample sizes. To assess the presence of landscape patterns of genetic diversity, we tested the relationship between genetic diversity metrics and environmental (i.e., latitude, longitude, and elevation) and physiological (i.e., thermal thresholds) factors using a nonparametric Spearman rank-order correlation coefficient test [100]. Comparisons of the environmental factors were made at the individual level, whilst physiological associations were investigated at the population level.

A minimum number of individuals per site was set for population genetic analyses. Genetic diversity metrics were calculated for sites with four or more individuals, and pairwise *F_ST_* was computed between sites with at least five individuals. After SNP filtering steps, sites for some species had less than the required number of individuals for analyses, and, as such, reasonable statistical values could not be computed for all ten species.

### 4.5. Modelled Current and LGM Potential Distributions for Three Focal Species

We sought to investigate the historical drivers behind the observed genetic patterns for three focal species: *Astelia alpina, Richea continentis*, and *Epacris paludosa*. The potential distributions were modelled under both current interglacial and paleoclimate glacial conditions and compared to identify whether climatic extremes facilitated the distributional dynamics that culminated in distinct genetic signatures. As an exploratory approach to look at broad-scale distributions, potential distributions were defined here as areas that were environmentally favourable, i.e., represented suitable habitat, for the species at a given time.

#### 4.5.1. Floristic Occurrence Data

The focal species selected showed contrasting genetic patterns that were generally representative of different patterns observed for study species (e.g., significance of correlations with F_ST_ and elevation and/or distance and comparative genetic diversity; Table S5). Occurrence records for each species were downloaded from the Atlas of Living Australia database [69]. Floristic data were cleaned retaining records based on date (1980–2023), institution (e.g., herbarium and government department), and georeferencing precision (up to 1 km). Geographically doubtful records, e.g., falling in the ocean, were removed, and duplicate records, e.g., from the exact same reference and locality description, were filtered.

#### 4.5.2. Current and LGM Extents

Study extents were delimited for both the present and LGM conditions and set as the geographic area where the focal species could potentially occur. For each species, the current extent was delimited by mapping its occurrence points on a bioregional classification of terrestrial Australia and selecting the bioregions where points were found. To do so, we relied on the Interim Biogeographic Regionalisation for Australia (IBRA; [101]) that categorises Australia’s landscapes into geographically distinct bioregions considering common climate, geology, landform, native vegetation and species information (Table S6). We assumed that the geographic, biotic, and abiotic specificities of each bioregion would emulate a fitted environmental envelope for the species’ potential distribution.

Once the present extent was delimited as the sum of all bioregions where the species currently occur, we indirectly transferred the environmental envelope to LGM conditions, assuming niche conservatism. Because there are no bioregional classifications of Australia for the LGM, we characterised the environmental envelope using two bioclimatic proxies: monthly mean daily temperature (*tas*) and monthly precipitation amount (*pr*). Both are acknowledged to be the main drivers of plant distribution worldwide [3,102] and in eastern Australia, where they are key to explaining latitudinal and altitudinal (temperature), as well as longitudinal patterns (precipitation). To capture a clear bioclimatic signal for our environmental envelope, we used the two variables as their mean value and standard deviation, with the latter to account for seasonality and variability. We also investigated annual temperature (bio1) and annual precipitation (bio12); however, we found that these ranges under current conditions did not as closely represent the present-day bioregion spread of each species as well as *tas* and *pr*.

There were several steps in generating these LGM environmental envelopes. First, we downloaded the monthly datasets of *tas* and *pr* at 30 arc s and for the period 1981–2010 from the CHELSA V2.1 database [103,104] and computed four annual variables as the mean and standard deviation of respective monthly datasets. We then cropped the four corresponding rasters to the current extent of each species and extracted the value ranges of each variable. Second, we downloaded the *tas* and *pr* variables at 30 arc s from PMIP3 datasets provided by the CHELSA V1.2 database [105]. After the four annual variables were extracted for the LGM, each raster was cropped to the respective variable ranges and stacked, retaining only the areas where all four variables coincided. To incorporate the variation between predicted climate scenarios and reduce prediction uncertainty, four global climate models (GCMs) were selected to represent paleoclimatic conditions: CCSM4, CNRM-CM5, FGOALS-g2, and MRI-CGCM3. The preferred GCMs had performance scores above the mean for south-eastern Australia based on the ability of each scenario to simulate conditions across the Australian continent [106]. Because ice cover is thought to have been less than 15 km^2^ on mainland Australia during this period [107], to simplify the LGM study extent, we chose to ignore the glacier surface when projecting from the current extent.

#### 4.5.3. Model Preparation

Once the species-specific study extents were defined, pseudo-absences were drawn to complement the presence data. Following the advice from Barbet-Massin et al. [108], a large number of pseudo-absences were generated (set as double the number of presences for each species; *A. alpina* had 110 presences and 220 absences; *R. continentis* had 203 presences and 400 absences; and *E. paludosa* had 1178 presences and 3500 absences) and randomly scattered on the study extent, provided they fell in grid cells without presence points (randomPoints function in R package dismo; [109]).

To serve as predictors for the species’ current and paleodistribution, 19 bioclimatic variables were retrieved from the CHELSA V2.1 database [103,104]. First, we downloaded the full variable set at 30 arc s for the current conditions (1981–2010) and adjusted them to the current study extent. To check for multicollinearity between variables and reduce redundancy, we performed a variance inflation factor correlation analysis (vif function in the R package usdm; [110]) and retained all variables with a VIF < 3 [111]. The vif selected the following variables depending on the current extent of each focal species: (i) for *A. alpina*—isothermality (bio3), temperature seasonality (bio4), mean daily air temperatures of the wettest quarter (bio8), mean daily air temperatures of the driest quarter (bio9), precipitation amount of the driest month (bio14) and precipitation seasonality (bio15); (ii) for *R. continentis*—mean annual air temperature (bio1), bio3, bio4, bio9, bio15, and mean monthly precipitation amount of the warmest quarter (bio18); and (iii) for *E. paludosa*—bio3, bio4, bio9, mean daily air temperatures of the warmest quarter (bio10), bio14, and bio15. Finally, the selected predictors were obtained for LGM conditions for the four GCMs previously identified (CCSM4, CNRM-CM5, FGOALS-g2, and MRI-CGCM3). The vif-selected paleobioclimatic variables were downloaded from the CHELSA V2.1 PMIP3 dataset [105] and cropped to the previously delimited LGM extent.

#### 4.5.4. Species Distribution Models

We determined the potential distributions of focal species under current and LGM conditions by developing a series of species distribution models (SDMs). SDMs were run for each species using their specific bioclimatic predictor set and two complementary algorithms: Generalised Linear Models (GLMs) and Random Forests (RFs). We chose these two different algorithms to explore the relationship between the response variable (distribution data as presence and pseudo-absence points) and explanatory variables (the predictors) under both a flexible linear Gaussian-identity distribution-link approach (GLM) and decision trees with classification and regression approaches (RF). Individual models were run 100 times (repeated 50 times per algorithm), randomly and repeatedly selecting 75% of the distribution data as the training set, and 25% as the testing dataset (R package biomod2; [112]). For prediction accuracy, the true skill statistic (TSS) was used where the models with a TSS value higher or equal to 0.6 were retained and ensembled [113], an appropriate approach used under similar modelling situations [114].

The ensemble models were projected onto the current predictor set as well as every GCM predictor set to obtain the final probabilistic predictions for current and LGM conditions. The probabilistic projections were binarised using a threshold approach that optimises the sensitivity and specificity metrics under the *optimal.threshold* function in R package ecospat [115]. This method maximises the sum of the sensitivity and specificity [116,117]. In other words, a high sensitivity means that the model predicts a high number of true presences and a low number of false presences, whereas a high specificity means the model predicts a high number of true absences and few false absences. To determine the LGM suitable habitat for each species, the binary models for the four GCMs were overlapped, and the areas where at least two scenarios coincided were retained. Final consensus maps were developed by overlaying current and paleoclimate distributions for each species. The total area of suitable habitat for each time period (current and LGM) and the overlapping stable habitat were calculated as land coverage using the freq function under the R package terra [118].

## Figures and Tables

**Table 1:** Linear mixed model results for the influence of the effect of elevation on thermal tolerance thresholds of alpine plant species: critical cold threshold (T_crit-cold_), critical heat threshold (T_crit-hot_), and thermal threshold breadth (TTB). Models for all thermal thresholds included elevation as the fixed effect and species as a random effect. Additionally, the T_crit-cold_ model included date as a random effect and the T_crit-hot_ model included site as a random effect. Model results for the marginal R^2^ (marg. R^2^; elevation without random effects) and conditional R^2^ (cond. R^2^_;_ both elevation and random effects of species and date or site) on thermal thresholds. Significance level at 0.05 α.

Factor	T_crit-cold_		T_crit-hot_		TTB	
	F _(1, 44.5)_	*p*-Value	F _(1, 25.2)_	*p*-Value	F _(1, 512.7)_	*p*-Value
Elevation	0.289	0.593	1.72	0.097	0.723	0.396
Random effects of species and date or site	Marg. R^2^ (elevation only)	Cond. R^2^ (elevation + species and date)	Marg. R^2^ (elevation only)	Cond. R^2^ (elevation + species and site)	Marg. R^2^ (elevation only)	Cond. R^2^ (elevation + species)
	0.001	0.692	0.006	0.355	0.001	0.450

**Table 2:** Linear mixed model results for species effect on thermal tolerance thresholds: critical cold threshold (T_crit-cold_), critical heat threshold (T_crit-hot_), and thermal tolerance breadth (TTB) for ten alpine species. Linear mixed models for T_crit-cold_ include date as a random effect and models for T_crit-hot_ include site as a random effect. The model mean and standard error for each species T_crit-cold_, T_crit-hot_, and TTB were extracted from estimated marginal means’ post hoc analyses. Species are sorted from the widest to the narrowest TTB; * indicates significance level at 0.05 α.

Factor	T_crit-cold_ (°C)		T_crit-hot_ (°C)		TTB (°C)	
	F _(9, 521.1)_	*p*-Value	F _(9, 578.5)_	*p*-Value	F _(9, 508)_	*p*-Value
Species	79.0	<0.0001 *	32.3	<0.0001 *	37.0	<0.0001 *
	Mean ± SE		Mean ± SE		Mean ± SE	
*Hovea montana*	−17.8 ± 0.5		40.6 ± 0.6		60.6 ± 1.0	
*Grevillea australis*	−10.8 ± 0.4		50.5 ± 0.6		60.2 ± 0.7	
*Astelia alpina*	−13.5 ± 0.4		44.3 ± 0.6		58.3 ± 0.7	
*Epacris paludosa*	−11.6 ± 0.3		45.1 ± 0.5		56.7 ± 0.6	
*Oxylobium ellipticum*	−14.8 ± 0.4		41.7 ± 0.6		56.4 ± 0.7	
*Richea continentis*	−10.6 ± 0.3		44.2 ± 0.6		54.9 ± 0.6	
*Tasmannia xerophila*	−8.5 ± 0.4		46.5 ± 0.7		54.8 ± 0.7	
*Aciphylla glacialis*	−7.7 ± 0.4		41.6 ± 0.6		49.4 ± 0.6	
*Prostanthera cuneata*	−9.6 ± 0.4		39.5 ± 0.6		49.2 ± 0.7	
*Psychrophila introloba*	−6.4 ± 0.4		41.9 ± 0.7		48.2 ± 0.8	

**Table 3:** Alpine study plant species that span taxonomic families and growth forms characteristics of the Australian Alps Bioregion. Sampling was conducted across a broad elevation range to assess thermal tolerance (within Kosciuszko National Park) and genetic patterns (across south-eastern Australia). Refer to Figure 5 for the sampling and distributional range for each species. The asterisks (*) indicate the subset of seven species that were sampled across their wider distributional ranges across south-eastern Australia.

Species	Family	Growth Form	Elevation Range of Sampling (m a.s.l)	
			Thermal Tolerance	Genetic
*Aciphylla glacialis* (F.Muell.) Benth.	Apiaceae	Forb	1735–2066	1724–2058
*Astelia alpina* R.Br. *	Asteliaceae	Forb	1735–2044	1651–2058
*Psychrophila introloba* (F.Muell.) W.A.Weber	Ranunculaceae	Forb	1735–2044	1716–2080
*Richea continentis* B.L.Burtt *	Ericaceae	Shrub	1502–2066	1483–2057
*Epacris paludosa* R.Br. *	Ericaceae	Shrub	1503–2070	1449–2034
*Grevillea australis* R.Br. *	Proteaceae	Shrub	1503–2070	1363–1994
*Prostanthera cuneata* Benth.	Lamiaceae	Shrub	1503–1966	1364–1993
*Hovea montana* (Hook.f.) J.H.Ross *	Fabaceae	Shrub	1420–1894	1365–1902
*Oxylobium ellipticum* (Vent.) R.Br. *	Fabaceae	Shrub	1411–1933	1366–1935
*Tasmannia xerophila* M.Gray *	Winteraceae	Shrub	1420–1920	1221–1796

## Data Availability

All data will be made available on an open source database upon acceptance of the manuscript.

## Supplementary Materials

The following supporting information can be downloaded at: https://www.mdpi.com/article/10.3390/plants13091271/s1, Table S1: Thermal tolerance study sites across three elevation gradients in Kosciuszko National Park of south-east New South Wales, Australia. Key sites where microsite logging stations (logging leaf and air temperature) were established are marked with asterisks (*). For each elevation gradient, sites are listed in ascending order based on elevation; Table S2: Model fitting process implemented during elevation thermal gradient statistical analysis. The model incorporated a response variable of daily mean minimum temperature (Tmin; continuous) or daily mean maximum temperature (Tmax; continuous) and fixed explanatory variable of elevation (continuous). Best model fit was determined based on three components: lowest Akaike Information Criterion (AIC) value, significance between models and the level of variance explained by random effects. Final model selected includes random effect of date (categorical, 3 levels) for both response variables. Linear mixed models fit with R package lme4 (Bates et al., 2015). Final models selected are shown in bold. Superscripts indicate an incomplete model fit due to singular fits.
