# Supplementary material for: Environmental and Biogeographic Drivers behind Alpine Plant Thermal Tolerance and Genetic Variation
Source: Plants (Basel). 2024 May 4;13(9):1271. doi: 10.3390/plants13091271 (PMC11085172; doi:10.3390/plants13091271)
Supplement: Supplementary file 1 [file plants-13-01271-s001.zip › plants-2876149-supplementary.pdf]

Supporting information

Environmental and biogeographic drivers behind alpine plant thermal tolerance and genetic variation on a local scale

Table S1. Thermal tolerance study sites across three elevation gradients in Kosciuszko National Park of south-east New South Wales, Australia. Key sites where microsite logging stations (logging leaf and air temperature) were established are marked with asterisks (*). For each elevation gradient, sites are listed in ascending order based on elevation.

Site	Latitude (°)	Longitude (°)	Elevation (m a.s.l)	Aspect
**Charlotte Pass**				
Pipers Creek *	-36.3927	148.4356	1636	S
Snowy River (low) *	-36.4306	148.3218	1735	NW
Charlotte Pass ski hill *	-36.4355	148.3279	1811	NE
Charlotte Pass carpark	-36.4346	1483270	1871	NE
Blue Lake walk	-36.4223	148.3147	1876	S
Stillwell snow patch *	-36.4420	148.1483	1966	S
Blue Lake lookout *	-36.4092	148.3065	1972	SE
**Thredbo**				
Merritts nature trail (low) *	-36.5002	148.3047	1417	SE
Merritts traverse *	-36.4918	148.2951	1740	SE
Merritts nature trail (high)	-36.4939	148.2889	1894	SE
Thredbo River *	-36.4894	148.2839	1959	SE
Kosciuszko walk	-36.4839	148.2820	2019	S
Kosciuszko lookout *	-36.4801	148.2767	2059	S
**Perisher**				
Munyang River *	-36.3379	148.4020	1503	E
Rainbow Lake *	-36.3700	148.4759	1598	NE
Porcupine Rocks (low) *	-36.4157	148.4081	1773	N
Porcupine Rocks (high) *	-36.4291	148.3980	1903	W

Table S2. Model fitting process implemented during elevation thermal gradient statistical analysis. The model incorporated a response variable of daily mean minimum temperature (T_{min}; continuous) or daily mean maximum temperature (T_{max}; continuous) and fixed explanatory variable of elevation (continuous). Best model fit was determined based on three components: lowest Akaike Information Criterion (AIC) value, significance between models and the level of variance explained by random effects. Final model selected includes random effect of date (categorical, 3 levels) for both response variables. Linear mixed models fit with R package *lme4* (Bates et al., 2015). Final models selected are shown in bold. Superscripts indicate an incomplete model fit due to singular fits.

**Model**	**T_{min} AIC**	**T_{max} AIC**
Basic model		
Response ~ Elevation	150.4	234.4
Test random effects of date, to account for weather differences among sampling days, site and site nested within transect, to account for the three elevation gradients; local effects of site aspect and slope; and the spatial grouping of sites within the three elevation gradients		
Response ~ Elevation + (1∣Date)	**152.3**	**200.5**
Response ~ Elevation + (1∣Site)	164.7 ^{*s}	244.5 ^{*s}
Response ~ Elevation + (1∣Transect)	164.7 ^{*s}	244.5 ^{*s}
Response ~ Elevation + (1∣ Transect/Site)	166.7 ^{*s}	246.5 ^{*s}

Table S3. Three-step model fitting process implemented during thermal threshold statistical analysis. The model incorporated a response variable of thermal tolerance thresholds: critical cold thresholds ($T_{crit\text{-}cold}$; continuous), critical heat thresholds ($T_{crit\text{-}hot}$; continuous) or thermal tolerance breadth (TTB; continuous) and fixed explanatory variable of elevation (continuous). Best model fit was determined based on three components: lowest Akaike Information Criterion (AIC) value, significance between models and the level of variance explained by random effects. Final model selected from step 3 includes random intercept of species (categorical, 10 levels) for all three response variables. The $T_{crit\text{-}cold}$ model also included date of sampling (categorical, 8 levels) as an additional random effect and $T_{crit\text{-}hot}$ model also included site (categorical, 17 levels) as an additional random effect. Linear mixed models fit with R package *lme4* (Bates et al., 2015). Best models selected for each step are shown in bold. Superscripts indicate an incomplete model fit, either convergence issues (c) or singular fit (s).

**Model**	**$T_{crit\text{-}cold}$ AIC**	**$T_{crit\text{-}hot}$ AIC**	**TTB AIC**
Basic model			
Response ~ Elevation	2918.5	3859.5	3394.2
Step 1. Test random intercepts and random slopes for elevation within each level of species			
Response ~ Elevation + (Elevation∣Species)	2525.5 *[c]	3674.4 *[s]	3196.7 *[c]
Response ~ Elevation + (1∣Species)	**2521.7**	**3673.1**	**3194.2**
Step 2. Test random effects of date, to account for weather differences among sampling days, site and site nested within transect, to account for the three elevation gradients; local effects of site aspect and slope; and the spatial grouping of sites within the three elevation gradients			
Response ~ Elevation + (1∣Species)	**2521.7**	**3673.1**	**3194.2**
Response ~ Elevation + (1∣Date)	2916.2	3870.7	3401.8
Response ~ Elevation + (1∣Site)	2930.8	3872.2	3403.8
Response ~ Elevation + (1∣Transect)	2934.3 *[s]	3872.2	3407.3
Response ~ Elevation + (1∣ Transect/Site)	2932.8 *[s]	3873.4	3405.8
Step 3. Test combinations of random effects			
Response ~ Elevation + (1∣Species)	2521.7	3673.1	**3194.2**
Response ~ Elevation + (1∣Species) + (1∣Date)	**2513.8**	3672.5	3195.9
Response ~ Elevation+ (1∣Species) + (1∣Site)	2521.0	**3671.8**	3196.2 *[s]
Response ~ Elevation + (1∣Species) + (1∣Transect)	2523.7 *[s]	3675.0	3196.2 *[s]
Response ~ Elevation + (1∣Species) + (1∣ Transect/Site)	2523.0 *[s]	3673.8 *[s]	3198.2 *[s]
Response ~ Elevation+ (1∣Species) + (1∣Site) + (1∣Date)	2515.6	3673.6	3197.9 *[s]

Table S4. Model fitting process implemented during thermal threshold statistical analysis. The model incorporated a response variable of thermal tolerance thresholds: critical cold thresholds ($T_{crit-cold}$; continuous), critical heat thresholds ($T_{crit-hot}$; continuous) or thermal tolerance breadth (TTB; continuous) and fixed explanatory variable of species (categorical, 10 levels). Best model fit was determined based on three components: lowest Akaike Information Criterion (AIC) value, significance between models and the level of variance explained by random effects. Final model selected includes random intercepts of date of sampling (categorical, 8 levels) for the $T_{crit-cold}$ model, site (categorical, 17 levels) for the $T_{crit-hot}$ model and no random effects for the TTB model. Linear mixed models fit with R package *lme4* (Bates et al., 2015). Final models selected are shown in bold. Superscripts indicate an incomplete model fit due to singular fits.

**Model**	**$T_{crit-cold}$ AIC**	**$T_{crit-hot}$ AIC**	**TTB AIC**
Basic model			
Response ~ Species	2475.6	3638.9	**3154.3**
Step 1. Test random effects of date, to account for weather differences among sampling days, site and site nested within transect, to account for the three elevation gradients; local effects of site aspect and slope; and the spatial grouping of sites within the three elevation gradients			
Response ~ Species + (1 ǀ Date)	**2466.1**	3630.8	3144.8 ^{*s}
Response ~ Species + (1 ǀ Site)	2474.9	**3628.6**	3144.8 ^{*s}
Response ~ Species + (1 ǀ Transect)	2481.4 ^{*s}	3633.5 ^{*s}	3144.8 ^{*s}
Response ~ Species + (1 ǀ Transect/Site)	2476.9 ^{*s}	3630.6 ^{*s}	3146.8 ^{*s}
Step 2. Test combinations of random effects			
Response ~ Species + (1 ǀ Date) + (1 ǀ Site)	2468.0	3630.6	3146.8 ^{*s}

Table S5. Focal species selected to perform species distribution models. Species were selected based on presence of correlations between genetic differentiation (F_{ST}) and elevation or distance (ns: non-significant; s: significant) and range of inbreeding coefficient (F_{is}) within Kosciuszko National Park (KNP) and across south-eastern Australia. Genetic diversity of each species is considered high or low relative to the other study species. Lower values of F_{is} indicate higher genetic diversity and higher values of F_{is} indicate lower genetic diversity.

Species	F_{ST} correlations			F_{is} range		Genetic diversity
	KNP		SE Australia	KNP	SE Australia	
	Elevation	Distance	Distance			
Astelia alpina	ns	s	s	0.015 – 0.163	0.002 – 0.160	High
Richea continentis	s	ns	s	0.181 – 0.405	0.192 – 0.418	Low
Epacris paludosa	ns	ns	s	0.288 – 0.351	0.182 – 0.338	Low

Table S6. Interim Biogeographic Regionalisation for Australia (IBRA) bioregions and climatic variables used to delimit current and last glacial maximum study areas for three focal species: *Astelia alpina*, *Richea continentis* and *Epacris paludosa*. Mean and standard deviation (SD) of monthly temperature and precipitation variables were calculated.

**Species**	**IBRA bioregions**	**Mean daily temperature range (tas; °C)**		**Monthly precipitation amount range (pr; kg m^{-2} $month^{-1}$)**	
		**Mean**	**SD**	**Mean**	**SD**
Astelia alpina	Australian Alps (AUA)<br>Ben Lomond (BEL)<br>Tasmania Central Highlands (TCH)<br>Tasmania Southern Ranges (TSR)<br>Tasmania West (TWE)	1.9 – 13.2	1.5 – 5.6	4.7 – 26.2	0.7 – 6.7
Richea continentis	Australian Alps (AUA)<br>South East Corner (SEC)<br>South Eastern Highlands (SHE)	2.8 – 16.4	2.3 – 5.9	3.8 – 15.5	0.7 – 5.3
Epacris paludosa	Australian Alps (AUA)<br>Furneaux (FUR)<br>NSW North Coast (NNC)<br>South East Corner (SEC)<br>South Eastern Highlands (SHE)<br>Sydney Basin (SYB)<br>Tasmania Northern Slopes (TNS)	2.8 – 20.0	2.1 – 5.9	3.8 – 18.5	0.6 – 7.4

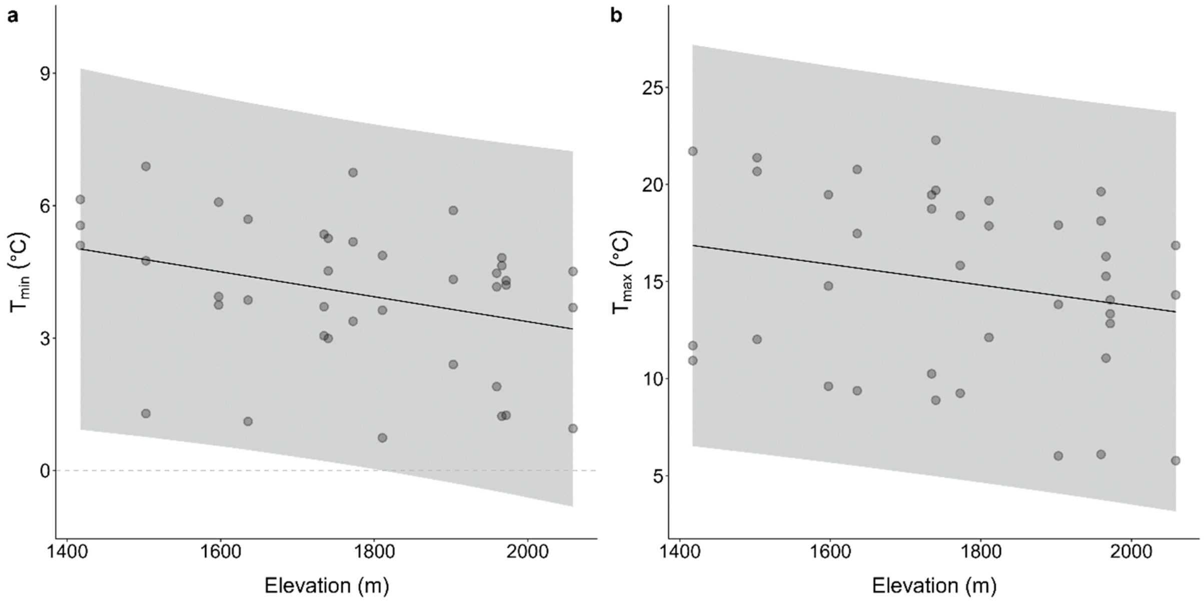

Figure S1. The relationship between elevation and ambient air temperature parameters: (a) mean minimum temperature (T_{min}) and (b) mean maximum temperature (T_{max}). Daily mean maximum and mean minimum temperatures were extracted from mean temperatures recorded every minute from a proceeding sampling frequency of five seconds. Ambient air temperature was measured over three consecutive days during the austral summer along three elevation gradients in Kosciuszko National Park, NSW. Circles represent observed daily mean maximum and mean minimum temperatures over the continuous recording period, with colours representing different temperature logging stations established along the gradients. The regression lines and confidence intervals are the predictions of the linear mixed model accounting for date as a random factor. Solid regression lines indicate that there was a significant relationship between elevation and air temperature and the grey ribbons represent 95% confidence intervals.

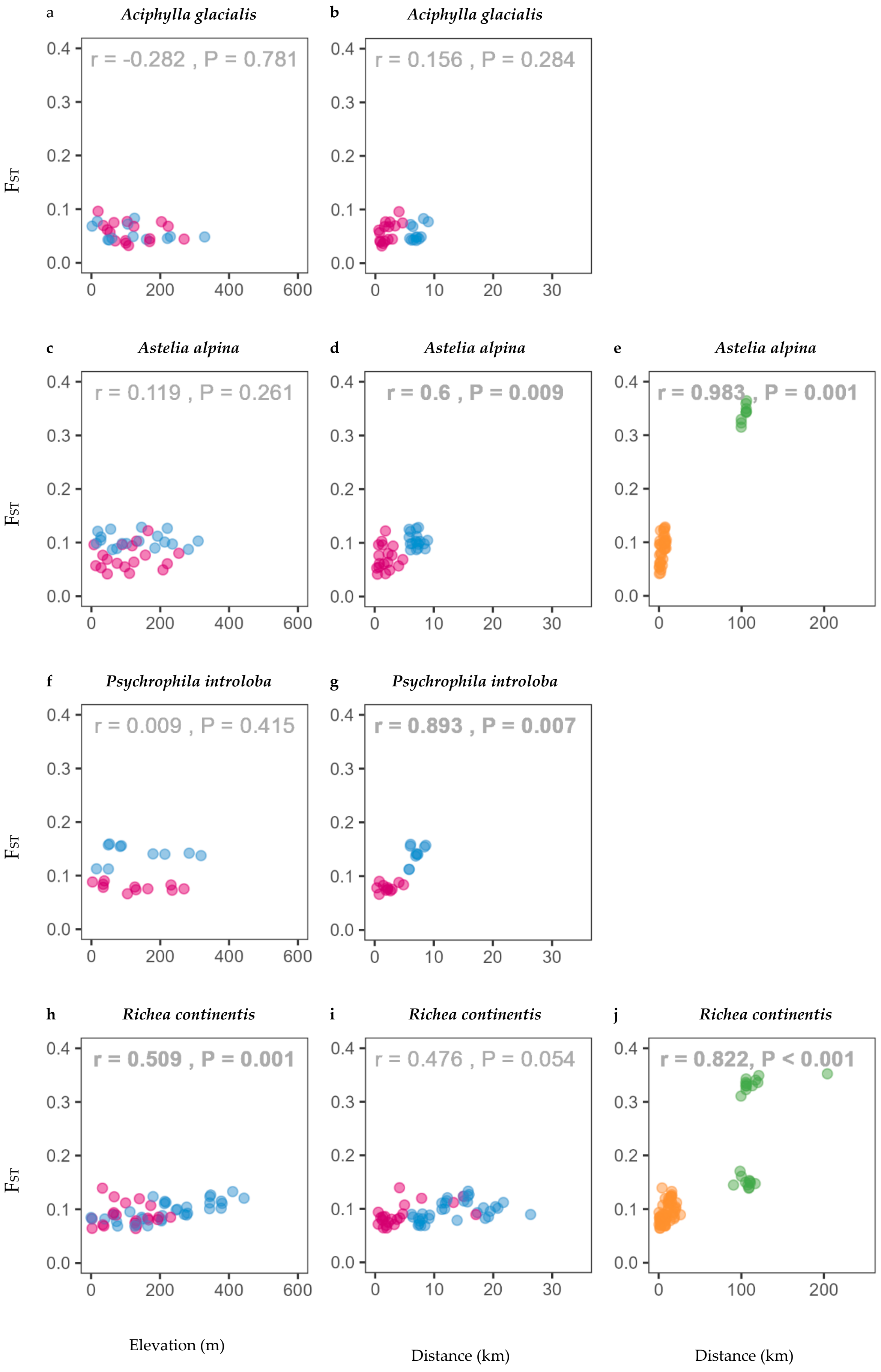
a
Aciphylla glacialis
r = -0.282 , P = 0.781
b
Aciphylla glacialis
r = 0.156 , P = 0.284
c
Astelia alpina
r = 0.119 , P = 0.261
d
Astelia alpina
r = 0.6 , P = 0.009
e
Astelia alpina
r = 0.983 , P = 0.001
f
Psychrophila introloba
r = 0.009 , P = 0.415
g
Psychrophila introloba
r = 0.893 , P = 0.007
h
Richea continentis
r = 0.509 , P = 0.001
i
Richea continentis
r = 0.476 , P = 0.054
j
Richea continentis
r = 0.822, P < 0.001
FST
Elevation (m)
Distance (km)
Distance (km)

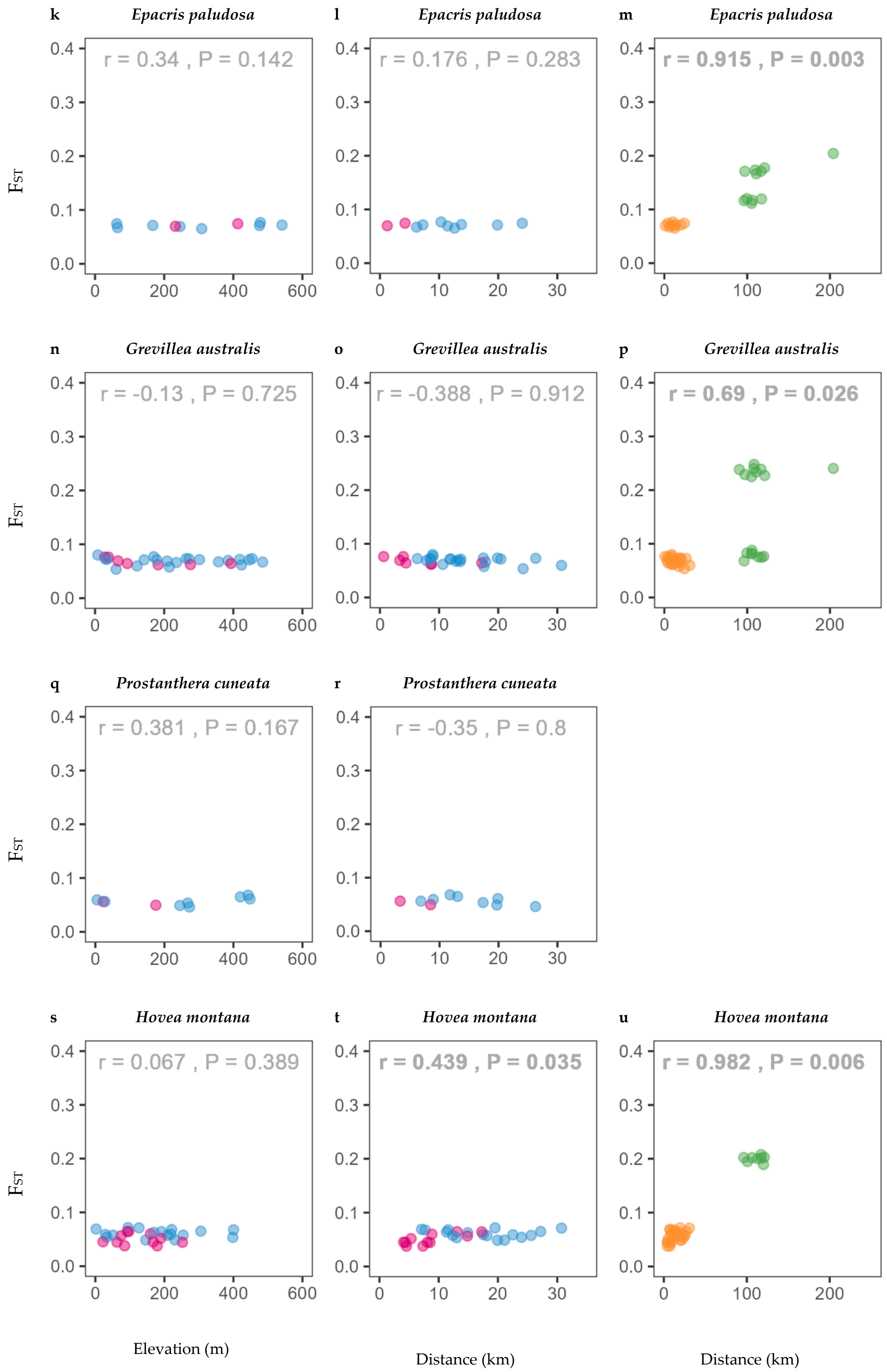

k
Epacris paludosa
r = 0.34 , P = 0.142
l
Epacris paludosa
r = 0.176 , P = 0.283
m
Epacris paludosa
r = 0.915 , P = 0.003
n
Grevillea australis
r = -0.13 , P = 0.725
o
Grevillea australis
r = -0.388 , P = 0.912
p
Grevillea australis
r = 0.69 , P = 0.026
q
Prostanthera cuneata
r = 0.381 , P = 0.167
r
Prostanthera cuneata
r = -0.35 , P = 0.8
s
Hovea montana
r = 0.067 , P = 0.389
t
Hovea montana
r = 0.439 , P = 0.035
u
Hovea montana
r = 0.982 , P = 0.006
FST
Elevation (m)
Distance (km)
Distance (km)

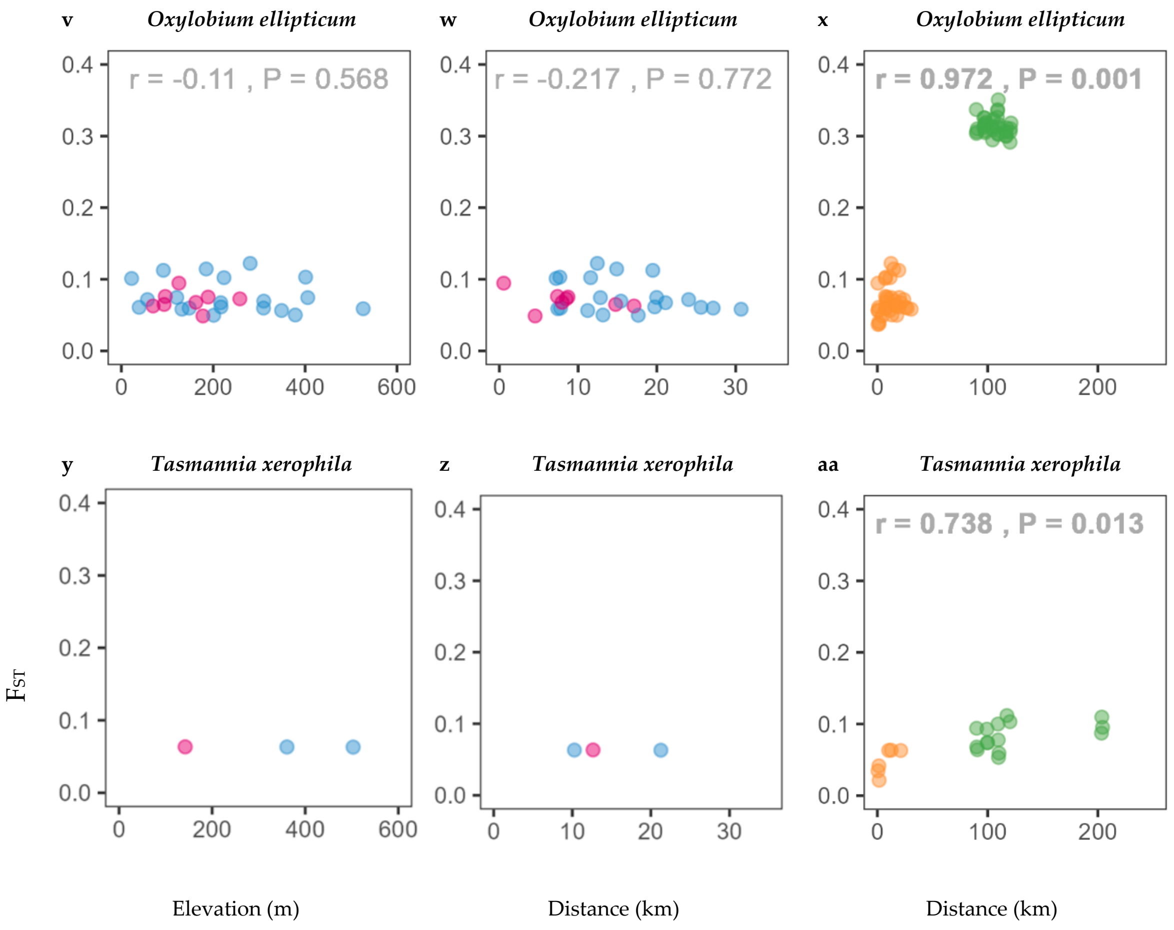

Figure S2. Isolation-by-distance plots comparing genetic differentiation (F_{ST}) of alpine plants across elevation in Kosciuszko National Park (left panels) and geographic distance in Kosciuszko National Park (middle panels) and across south-eastern Australia (right panels). For the plots in the left and middle panels, the coloured symbols represent within and between transect pairwise comparisons of sites (pink for within transects and blue for between transects). The study transects are Charlotte Pass, Perisher and Thredbo. For the plots in the right panel, the coloured symbols represent within and between region pairwise comparisons of sites (orange for within regions and green for between regions). The study regions are Namadgi, ACT, Kosciuszko National Park, NSW, and Alpine National Park, Victoria. Too few populations for *Tasmannia xerophila* in Kosciuszko National Park to compute reliable statistical metrics.

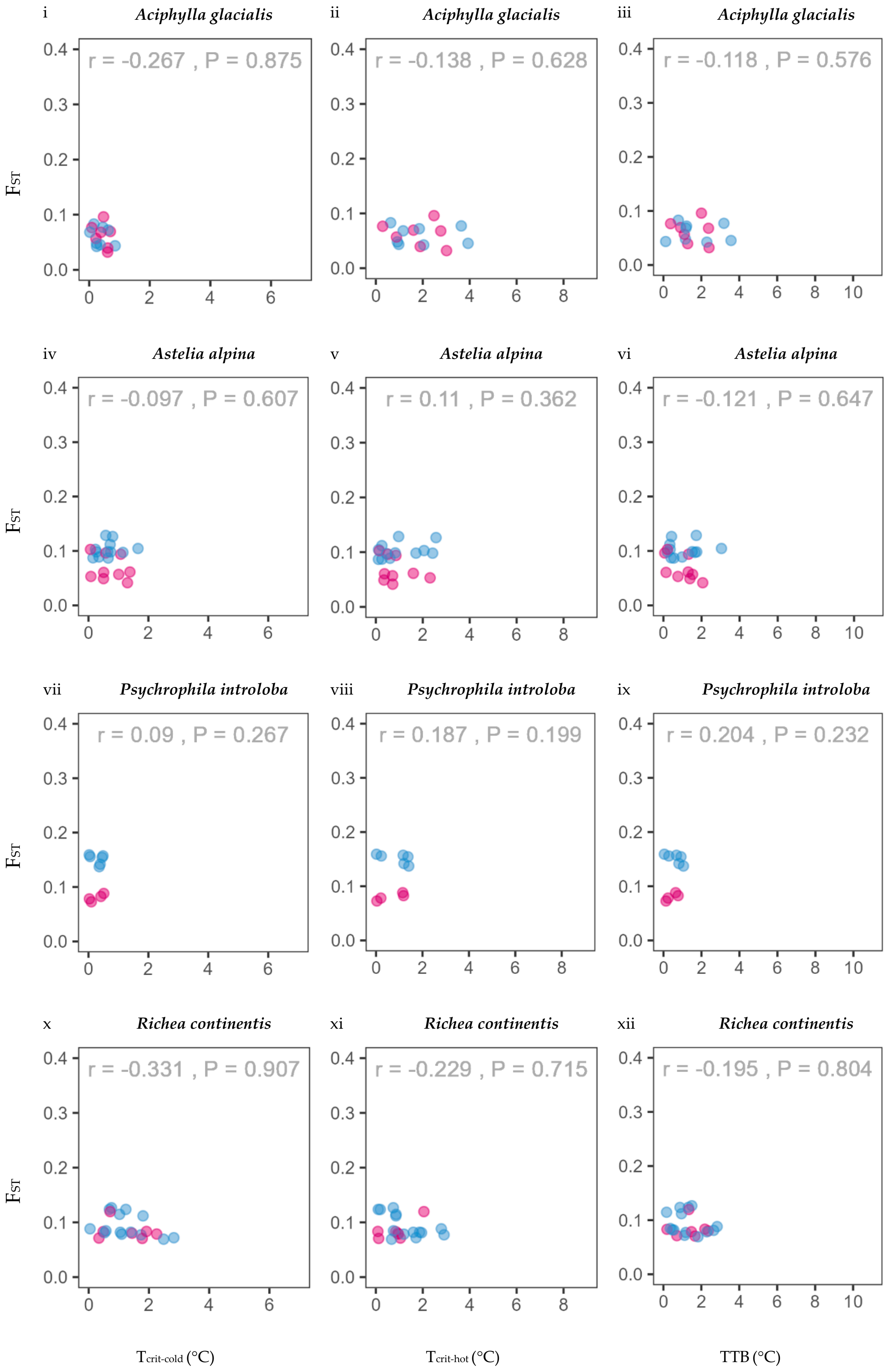
i Aciphylla glacialis
r = -0.267 , P = 0.875
ii Aciphylla glacialis
r = -0.138 , P = 0.628
iii Aciphylla glacialis
r = -0.118 , P = 0.576
iv Astelia alpina
r = -0.097 , P = 0.607
v Astelia alpina
r = 0.11 , P = 0.362
vi Astelia alpina
r = -0.121 , P = 0.647
vii Psychrophila introloba
r = 0.09 , P = 0.267
viii Psychrophila introloba
r = 0.187 , P = 0.199
ix Psychrophila introloba
r = 0.204 , P = 0.232
x Richea continentis
r = -0.331 , P = 0.907
xi Richea continentis
r = -0.229 , P = 0.715
xii Richea continentis
r = -0.195 , P = 0.804
FST
Tcrit-cold (°C)
Tcrit-hot (°C)
TTB (°C)

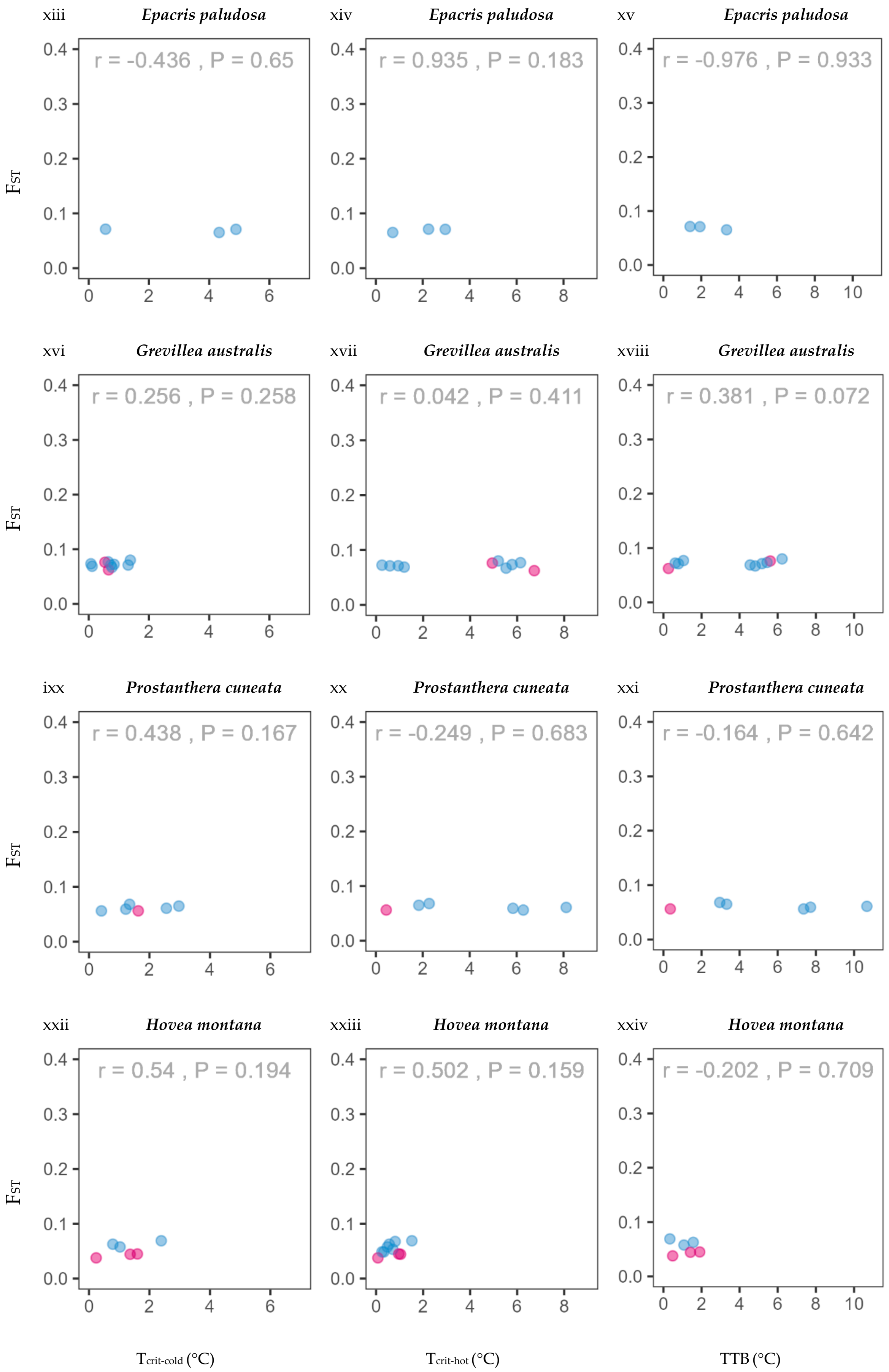
xiii Epacris paludosa
r = -0.436 , P = 0.65
xiv Epacris paludosa
r = 0.935 , P = 0.183
xv Epacris paludosa
r = -0.976 , P = 0.933
xvi Grevillea australis
r = 0.256 , P = 0.258
xvii Grevillea australis
r = 0.042 , P = 0.411
xviii Grevillea australis
r = 0.381 , P = 0.072
ixx Prostanthera cuneata
r = 0.438 , P = 0.167
xx Prostanthera cuneata
r = -0.249 , P = 0.683
xxi Prostanthera cuneata
r = -0.164 , P = 0.642
xxii Hovea montana
r = 0.54 , P = 0.194
xxiii Hovea montana
r = 0.502 , P = 0.159
xxiv Hovea montana
r = -0.202 , P = 0.709
FST
Tcrit-cold (°C)
Tcrit-hot (°C)
TTB (°C)

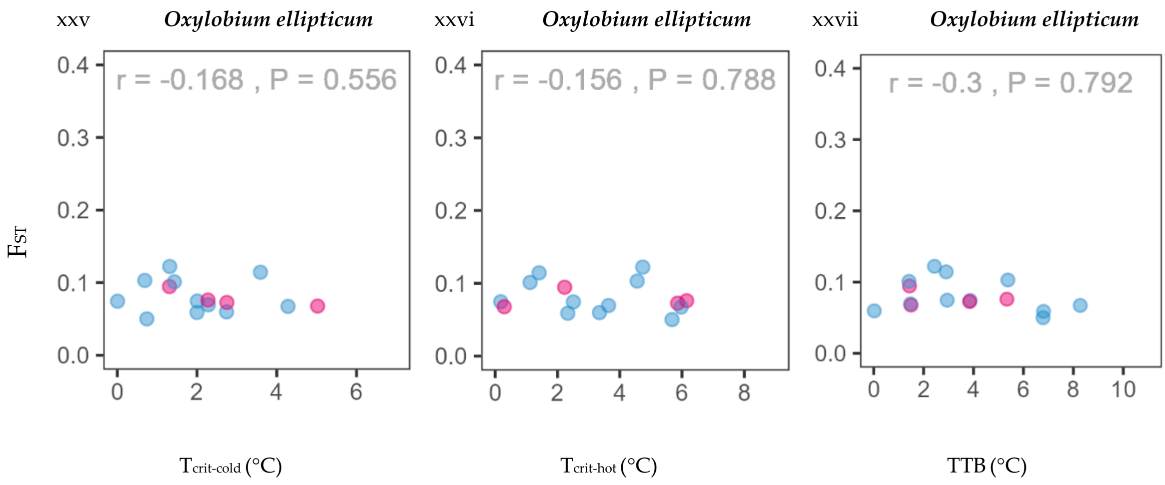

Figure S3. Isolation-by-distance plots comparing genetic differentiation (F_{ST}) of nine alpine plant species with cold thresholds ($T_{crit-cold}$: left panels), heat thresholds ($T_{crit-hot}$: middle panels), and thermal tolerance breadth (TTB: right panels) averaged per population in Kosciuszko National Park. The coloured symbols represent within and between transect pairwise comparisons of sites (pink for within transects and blue for between transects). The study transects are Charlotte Pass, Perisher and Thredbo. Only one paired site for *Tasmannia xerophila* (not analysed).

Table S7. Genetic diversity measures of ten alpine plant species from populations within Koscisuzko National Park (KNP) and representative populations across regions of south-eastern Australia in Namadgi National Park (ACT) and Alpine National Park (VIC). Genetic diversity measures are allelic richness (ar), expected heterozygosity (H_e), observed heterozygosity (H_o) and inbreeding coefficient (F_{is}). Sampling sites are ordered in ascending order of elevation within regions.

Species	Site	Latitude	Longitude	Elevation	*ar*	H_e	H_O	F_{is}
Aciphylla glacialis	**KNP**							
	Snowy River	-36.4291	148.3209	1738	1.595	0.223	0.193	0.093
	Snowy River 2	-36.4238	148.3153	1838	1.588	0.219	0.186	0.106
	Charlotte pass carpark	-36.4361	148.3252	1907	1.595	0.222	0.183	0.125
	Blue lake lookout	-36.4103	148.3058	1942	1.554	0.212	0.191	0.071
	Thredbo River	-36.4890	148.2839	1959	1.593	0.22	0.184	0.117
	Stillwell snow patch	-36.4426	148.3256	1961	1.562	0.214	0.189	0.087
	Wrights Creek	-36.4475	148.3288	2007	1.592	0.221	0.187	0.108
	Kosciusko lookout	-36.4803	148.2780	2067	1.571	0.221	0.169	0.177
	**Mean ± SE**				1.581 ± 0.006	0.219 ± 0.001	0.185 ± 0.003	0.111 ± 0.011
Astelia alpina	**KNP**							
	Snowy River	-36.4291	148.3209	1739	1.591	0.241	0.229	0.027
	Charlotte pass carpark	-36.4320	148.3273	1828	1.546	0.224	0.217	0.016
	Snowy River 2	-36.4245	148.3155	1836	1.584	0.236	0.226	0.022
	Blue lake lookout	-36.4100	148.3058	1947	1.593	0.24	0.224	0.045
	Stillwell snow patch	-36.4426	148.3257	1960	1.556	0.235	0.204	0.095
	Thredbo River	-36.4883	148.2834	1974	1.604	0.256	0.205	0.16
	Wrights Creek	-36.4483	148.3292	1993	1.538	0.225	0.19	0.12
	Kosciusko walk	-36.4843	148.2819	2020	1.628	0.256	0.236	0.052
	Kosciusko lookout	-36.4820	148.2794	2049	1.617	0.253	0.246	0.014
	**VIC**							
	Cope Hut site	-36.9082	147.2976	1654	1.523	0.225	0.224	0.002
	**Mean ± SE**				1.578 ± 0.011	0.239 ± 0.004	0.220 ± 0.005	0.055 ± 0.017
Epacris paludosa	**KNP**							
	Munyang	-36.3380	148.4021	1488	1.318	0.143	0.087	0.3
	Cascade trail	-36.5233	148.2643	1545	1.31	0.141	0.085	0.295
	Geehi	-36.2641	148.3740	1684	1.286	0.136	0.08	0.314
	Stillwell snow patch	-36.4425	148.3252	1755	1.278	0.133	0.073	0.338
	Charlottes Pass Ski Hill	-36.4357	148.3305	1794	1.316	0.143	0.088	0.286
	Thredbo River	-36.4890	148.2839	1961	1.307	0.141	0.079	0.334
	Wrights Creek	-36.4466	148.3286	2026	1.327	0.145	0.087	0.305
	**VIC**							
	Cope Hut site	-36.9081	147.2977	1666	1.301	0.132	0.093	0.222
	**ACT**							
	Ginini Flats	-35.5214	148.7842	1598	1.479	0.207	0.161	0.182

Species	Site	Latitude	Longitude	Elevation	*ar*	H_e	H_O	F_{is}
	**Mean ± SE**				1.325 ± 0.020	0.147 ± 0.008	0.093 ± 0.009	0.286 ± 0.017
Grevillea australis	**KNP**							
	Munyang	-36.3380	148.4023	1332	1.372	0.175	0.101	0.331
	Cascade trail	-36.5253	148.2649	1566	1.415	0.178	0.128	0.205
	Rainbow lake	-36.3700	148.4755	1621	1.277	0.142	0.091	0.28
	Geehi	-36.2640	148.3746	1688	1.351	0.168	0.102	0.3
	Porcupine rocks	-36.4166	148.4070	1782	1.348	0.167	0.103	0.293
	Charlottes Pass Ski Hill	-36.4355	148.3312	1787	1.352	0.153	0.113	0.172
	Snowy River 2	-36.4160	148.3109	1925	1.357	0.157	0.109	0.217
	Blue lake lookout	-36.4114	148.3071	1951	1.352	0.162	0.098	0.311
	Thredbo River	-36.4892	148.2842	1959	1.332	0.168	0.098	0.332
	Stillwell snow patch	-36.4434	148.3260	1989	1.379	0.168	0.108	0.273
	**VIC**							
	Cope Hut Site	-36.9088	147.2974	1676	1.378	0.172	0.113	0.263
	**ACT**							
	Ginini Flats	-35.5214	148.7842	1598	1.359	0.169	0.136	0.171
	**Mean ± SE**				1.356 ± 0.009	0.165 ± 0.003	0.108 ± 0.004	0.262 ± 0.017
Hovea montana	**KNP**							
	Meritts nature trail	-36.5022	148.3068	1386	1.458	0.198	0.154	0.16
	Munyang	-36.3405	148.4021	1532	1.457	0.192	0.144	0.177
	Cascade trail	-36.5255	148.2650	1565	1.442	0.19	0.143	0.177
	Aqueduct Track	-36.3729	148.4302	1594	1.41	0.189	0.12	0.275
	Rainbow lake	-36.3699	148.4756	1616	1.447	0.196	0.142	0.203
	Geehi	-36.2640	148.3747	1690	1.424	0.184	0.141	0.163
	Porcupine rocks	-36.4170	148.4068	1784	1.447	0.192	0.143	0.182
	Charlottes Pass Ski Hill	-36.4356	148.3311	1787	1.415	0.188	0.145	0.166
	Charlotte pass carpark	-36.4346	148.3266	1883	1.406	0.181	0.138	0.168
	**VIC**							
	Cope Hut Site	-36.9090	147.2971	1665	1.36	0.169	0.124	0.213
	**Mean ± SE**				1.427 ± 0.010	0.188 ± 0.003	0.139 ± 0.003	0.188 ± 0.011
Oxylobium ellipticum	**KNP**							
	Meritts nature trail	-36.5020	148.3066	1394	1.366	0.17	0.123	0.216
	Munyang	-36.3399	148.4021	1515	1.355	0.166	0.118	0.231
	Cascade trail	-36.5253	148.2648	1568	1.359	0.161	0.129	0.144
	Rainbow lake	-36.3699	148.4755	1610	1.328	0.163	0.093	0.355
	Geehi	-36.2645	148.3756	1704	1.351	0.167	0.101	0.319
	Porcupine rocks	-36.4162	148.4073	1776	1.337	0.161	0.107	0.264
	Charlottes Pass Ski Hill	-36.4359	148.3308	1795	1.3	0.143	0.095	0.257
	Charlotte pass carpark	-36.4369	148.3247	1920	1.348	0.163	0.105	0.28
	**ACT**							

Species	Site	Latitude	Longitude	Elevation	*ar*	H_e	H_O	F_{is}
	Mt Franklin Rd 3	-35.5042	148.7622	1541	1.283	0.139	0.088	0.282
	Ginini Flats	-35.5214	148.7842	1598	1.348	0.163	0.091	0.369
	Mt Franklin Rd 1	-35.5201	148.7659	1637	1.354	0.172	0.097	0.376
	Mt Franklin Road 2	-35.5251	148.7719	1666	1.299	0.148	0.088	0.332
	Upper Ginini Flats	-35.5265	148.7793	1670	1.359	0.17	0.101	0.334
	**Mean ± SE**				1.337 ± 0.007	0.160 ± 0.003	0.103 ± 0.004	0.289 ± 0.019
Prostanthera cuneata	**KNP**							
	Munyang	-36.3378	148.4015	1506	1.385	0.159	0.154	-0.005
	Geehi	-36.2648	148.3738	1681	1.431	0.18	0.163	0.053
	Porcupine rocks	-36.4157	148.4075	1773	1.335	0.153	0.122	0.12
	Charlottes Pass Ski Hill	-36.4354	148.3311	1789	1.356	0.152	0.137	0.042
	Snowy River 2	-36.4189	148.3129	1894	1.374	0.154	0.151	-0.015
	Charlotte pass carpark	-36.4371	148.3247	1924	1.35	0.155	0.115	0.179
	Blue lake lookout	-36.4113	148.3068	1949	1.371	0.154	0.139	0.054
	Thredbo River	-36.4895	148.2841	1953	1.374	0.158	0.138	0.065
	Stilwell snow patch	-36.4433	148.3259	1988	1.377	0.163	0.142	0.07
	**Mean ± SE**				1.373 ± 0.009	0.159 ± 0.003	0.140 ± 0.005	0.063 ± 0.020
Psychrophila introloba	**KNP**							
	Snowy River	-36.4294	148.3214	1726	1.4	0.16	0.103	0.267
	Snowy River 2	-36.4245	148.3155	1833	1.38	0.152	0.103	0.229
	Stilwell snow patch	-36.4422	148.3253	1959	1.353	0.148	0.089	0.307
	Blue lake lookout	-36.4090	148.3056	1962	1.368	0.149	0.091	0.297
	Wrights Creek	-36.4484	148.3294	1996	1.458	0.178	0.133	0.182
	Kosciusko walk	-36.4842	148.2820	2011	1.439	0.181	0.119	0.267
	Kosciusko lookout	-36.4818	148.2793	2045	1.44	0.18	0.12	0.262
	**Mean ± SE**				1.405 ± 0.015	0.164 ± 0.006	0.108 ± 0.006	0.259 ± 0.016
Richea continentis	**KNP**							
	Aqueduct Track	-36.3739	148.4306	1584	1.323	0.146	0.097	0.251
	Rainbow lake	-36.3701	148.4762	1617	1.269	0.13	0.07	0.363
	Geehi	-36.2638	148.3742	1684	1.293	0.138	0.077	0.339
	Porcupine rocks	-36.4146	148.4083	1757	1.282	0.133	0.075	0.325
	Charlottes Pass Ski Hill	-36.4356	148.3306	1796	1.307	0.141	0.085	0.289
	Charlotte pass carpark	-36.4321	148.3273	1831	1.302	0.142	0.08	0.337
	Snowy River 2	-36.4245	148.3154	1834	1.354	0.161	0.101	0.281
	Stillwell snow patch	-36.4427	148.3258	1961	1.336	0.151	0.101	0.244
	Thredbo River	-36.4889	148.2838	1961	1.302	0.146	0.085	0.317
	Blue lake lookout	-36.4091	148.3055	1964	1.354	0.153	0.111	0.192
	Wrights Creek	-36.4468	148.3285	2027	1.305	0.154	0.075	0.418
	Kosciusko lookout	-36.4819	148.2793	2040	1.268	0.131	0.085	0.253
	**VIC**							

Species	Site	Latitude	Longitude	Elevation	*ar*	H_e	H_O	F_{is}
	Cope Hut site	-36.9082	147.2977	1651	1.243	0.118	0.081	0.256
	**ACT**							
	Ginini Flats	-35.5214	148.7842	1598	1.356	0.155	0.111	0.217
	**Mean ± SE**				1.307 ± 0.009	0.143 ± 0.003	0.088 ± 0.004	0.292 ± 0.016
Tasmannia xerophila	**KNP**							
	Ngarigo campground	-36.4580	148.3858	1237	1.444	0.198	0.144	0.208
	Cascade trail	-36.5228	148.2643	1544	1.379	0.177	0.127	0.206
	Aqueduct Track	-36.3729	148.4301	1596	1.42	0.19	0.136	0.215
	Charlottes Pass Ski Hill	-36.4361	148.3309	1740	1.446	0.189	0.171	0.049
	Geehi	-36.2670	148.3792	1742	1.415	0.187	0.132	0.217
	**VIC**							
	Cope Hut site	-36.9090	147.2968	1664	1.442	0.21	0.138	0.278
	**ACT**							
	Ginini Flats	-35.5214	148.7842	1598	1.536	0.223	0.183	0.129
	Mt Franklin Rd 1	-35.5201	148.7659	1637	1.509	0.215	0.172	0.143
	Upper Ginini Flats	-35.5262	148.7797	1670	1.522	0.228	0.165	0.23
				**Mean ± SE**	1.457 ± 0.018	0.202 ± 0.006	0.152 ± 0.007	0.186 ± 0.023

Table S8. Results of non-parametric spearman rank correlation test (p-values plus rho in parenthesis) on the relationship between genetic diversity measures and latitude, longitude and elevation within Kosciuszko National Park (KNP) and across south-eastern Australia. Genetic diversity measures are allelic richness (*ar*), expected heterozygosity (H_e), observed heterozygosity (H_o) and inbreeding coefficient (F_{is}). Bold values indicate significant relationships.

**Species**	**Sampling range**	***ar***			H_e			H_o			F_{is}		
		**Latitude**	**Longitude**	**Elevation**	**Latitude**	**Longitude**	**Elevation**	**Latitude**	**Longitude**	**Elevation**	**Latitude**	**Longitude**	**Elevation**
Aciphylla glacialis	SE Australia	--	--	--	--	--	--	--	--	--	--	--	--
	KNP	0.629 (-0.204)	0.629 (0.204)	0.257 (-0.455)	0.548 (-0.252)	0.713 (0.156)	0.629 (-0.204)	0.139 (0.571)	0.352 (0.381)	0.289 (-0.429)	0.071 (-0.667)	0.493 (-0.286)	0.385 (0.357)
Astelia alpina	SE Australia	0.973 (-0.018)	0.218 (-0.430)	0.066 (0.612)	0.589 (-0.195)	0.125 (-0.518)	0.120 (0.524)	0.920 (0.036)	0.059 (-0.614)	0.776 (0.103)	0.946 (-0.030)	0.247 (0.406)	0.204 (0.442)
	KNP	0.291 (-0.400)	**< 0.001 (-9.67)**	0.213 (0.467)	0.160 (-0.510)	**0.002 (-0.879)**	0.242 (0.435)	0.982 (0.167)	**0.031 (-0.733)**	0.744 (0.133)	0.270 (-0.417)	0.644 (0.193)	0.552 (0.233)
Psychrophila introloba	SE Australia	--	--	--	--	--	--	--	--	--	--	--	--
	KNP	0.119 (-0.642)	0.819 (-0.107)	0.180 (0.571)	**0.036 (-0.786)**	0.294 (-0.464)	0.119 (0.643)	0.129 (-0.631)	0.788 (-0.126)	0.159 (0.595)	0.641 (0.216)	0.848 (-0.090)	0.670 (-0.198)
Epacris paludosa	SE Australia	0.879 (-0.071)	0.535 (0.286)	0.760 (0.143)	0.166 (0.504)	0.051 (0.664)	0.932 (0.034)	0.670 (0.198)	0.310 (0.450)	1.000 (0.000)	1.000 (0.000)	0.702 (-0.179)	0.589 (0.250)
	KNP	0.410 (0.317)	0.162 (0.517)	0.776 (-0.117)	0.877 (-0.073)	0.554 (0.273)	0.527 (0.291)	0.683 (0.159)	0.472 (0.276)	0.458 (-0.285)	0.880 (-0.067)	0.708 (-0.150)	0.230 (0.450)
Grevillea australis	SE Australia	0.313 (-0.319)	0.212 (-0.389)	0.442 (-0.245)	0.554 (-0.190)	0.363 (-0.289)	0.103 (-0.493)	0.476 (-0.228)	0.688 (-0.130)	0.415 (-0.260)	0.762 (0.098)	0.829 (-0.070)	0.485 (0.224)
	KNP	0.413 (-0.292)	0.192 (-0.450)	0.828 (-0.079)	0.506 (-0.239)	0.270 (-0.387)	0.506 (-0.239)	0.143 (-0.498)	0.336 (-0.340)	0.920 (-0.036)	0.293 (0.370)	0.829 (0.079)	0.881 (0.055)
Hovea montana	SE Australia	0.424 (0.286)	0.424 (0.286)	0.062 (-0.608)	0.590 (0.195)	0.235 (0.413)	0.053 (-0.626)	0.776 (-0.103)	0.751 (-0.116)	0.393 (-0.304)	0.947 (-0.024)	0.336 (0.340)	0.920 (-0.036)
	KNP	0.966 (0.017)	0.966 (0.017)	**0.035 (-0.703)**	0.781 (-0.109)	0.620 (0.192)	**0.038 (-0.695)**	0.262 (-0.418)	0.284 (-0.402)	0.306 (-0.385)	0.529 (0.243)	**0.032 (0.711)**	0.983 (0.008)
Oxylobium ellipticum	SE Australia	0.200 (0.753)	0.292 (-0.317)	0.266 (-0.333)	0.907 (0.036)	0.829 (0.066)	0.632 (-0.147)	**0.004 (-0.744)**	**0.007 (-0.708)**	0.667 (-0.132)	**0.003 (0.753)**	**< 0.001 (0.830)**	0.517 (0.198)
	KNP	0.420 (-0.333)	0.120 (-0.595)	**0.037 (-0.738)**	0.506 (0.277)	0.932 (-0.036)	0.143 (-0.566)	0.160 (-0.548)	0.102 (-0.619)	0.139 (-0.571)	0.086 (0.643)	0.058 (0.690)	0.183 (0.524)
Prostanthera cuneata	SE Australia	--	--	--	--	--	--	--	--	--	--	--	--
	KNP	0.404 (0.318)	0.932 (0.033)	0.683 (-0.159)	0.764 (0.117)	0.949 (0.025)	0.932 (0.033)	0.125 (0.550)	0.765 (0.117)	0.406 (-0.317)	0.244 (-0.433)	0.966 (-0.017)	0.244 (0.433)
Richea continentis	SE Australia	0.176 (0.383)	0.404 (0.242)	0.931 (0.025)	0.497 (0.198)	0.742 (0.097)	0.411 (0.239)	0.746 (0.095)	0.499 (-0.197)	0.737 (0.099)	0.852 (-0.055)	0.464 (0.213)	0.994 (0.002)
	KNP	0.957 (0.018)	0.519 (-0.207)	0.675 (0.135)	0.463 (-0.235)	0.230 (-0.375)	0.200 (0.398)	0.645 (-0.148)	0.055 (-0.565)	0.316 (0.317)	0.746 (0.105)	0.138 (0.455)	0.449 (-0.242)
Tasmannia xerophila	SE Australia	0.067 (0.633)	**0.0246 (0.733)**	0.732 (0.133)	0.139 (0.533)	**0.036 (0.700)**	0.932 (0.033)	0.077 (0.617)	0.067 (0.633)	0.765 (0.117)	0.433 (-0.300)	0.576 (-0.217)	0.668 (0.167)
	KNP	0.873 (0.100)	0.624 (0.300)	1.000 (0.000)	0.873 (0.100)	0.104 (0.800)	0.505 (-0.400)	0.873 (0.100)	0.624 (0.300)	1.000 (0.000)	0.188 (0.700)	0.285 (0.600)	0.624 (0.300)

Table S9. Results of non-parametric spearman rank correlation test (p-values plus rho in parenthesis) on the relationship between genetic diversity measures and cold thresholds ($T_{crit-cold}$), heat thresholds ($T_{crit-hot}$), and thermal tolerance breadth (TTB) averaged per population in Kosciuszko National Park. Genetic diversity measures are allelic richness (*ar*), expected heterozygosity (H_e), observed heterozygosity (H_o) and inbreeding coefficient (F_{is}). Bold values indicate significant relationships. Only one paired site for *Tasmannia xerophila* (not analysed).

**Species**	***ar***			***H_e***			***H_o***			***F_{is}***		
	**$T_{crit-cold}$**	**$T_{crit-hot}$**	**TTB**	**$T_{crit-cold}$**	**$T_{crit-hot}$**	**TTB**	**$T_{crit-cold}$**	**$T_{crit-hot}$**	**TTB**	**$T_{crit-cold}$**	**$T_{crit-hot}$**	**TTB**
Aciphylla glacialis	0.425 (0.406)	0.913 (-0.058)	0.913 (-0.058)	0.787 (0.143)	0.544 (-0.314)	0.544 (-0.314)	0.957 (-0.029)	0.329 (-0.486)	0.329 (-0.486)	0.957 (-0.029)	0.704 (0.200)	0.704 (0.200)
Astelia alpina	0.337 (-0.429)	0.119 (-0.643)	0.879 (-0.071)	0.878 (-0.072)	0.129 (-0.631)	0.504 (-0.306)	0.094 (-0.679)	0.337 (-0.429)	0.645 (0.214)	0.337 (0.429)	0.879 (0.071)	0.702 (-0.179)
Psychrophila introloba	0.873 (-0.100)	0.285 (0.600)	0.285 (0.600)	1.000 (0.000)	0.391 (0.500)	0.391 (0.500)	0.873 (-0.100)	0.285 (0.600)	0.285 (0.600)	0.741 (0.205)	0.434 (-0.462)	0.434 (-0.462)
Epacris paludosa	0.600 (-0.400)	0.200 (-0.800)	0.600 (0.400)	0.789 (-0.212)	0.262 (-0.738)	0.895 (0.105)	1.000 (0.000)	0.400 (-0.600)	0.800 (-0.200)	1.000 (0.000)	0.400 (0.600)	0.800 (0.200)
Grevillea australis	0.159 (0.595)	0.531 (-0.288)	0.558 (-0.270)	0.848 (-0.090)	0.969 (0.018)	0.908 (0.054)	0.193 (0.556)	0.531 (0.288)	1.000 (0.000)	0.432 (-0.357)	0.819 (-0.107)	0.819 (-0.107)
Hovea montana	0.434 (0.462)	0.148 (-0.667)	0.219 (-0.667)	0.805 (0.154)	0.084 (-0.754)	0.089 (-0.821)	0.747 (-0.200)	0.544 (-0.314)	0.624 (-0.300)	0.505 (0.400)	0.623 (-0.257)	0.391 (-0.500)
Oxylobium ellipticum	0.787 (0.143)	0.0724 (-0.771)	0.208 (-0.600)	0.700 (-0.203)	0.173 (-0.638)	0.354 (-0.464)	0.329 (0.486)	0.072 (-0.771)	**0.042 (-0.829)**	0.397 (-0.429)	0.072 (0.771)	**< 0.001 (1.00)**
Prostanthera cuneata	0.819 (-0.107)	0.879 (0.071)	0.432 (0.357)	0.939 (-0.036)	0.7017 (0.179)	0.253 (0.500)	0.482 (-0.321)	0.760 (0.143)	0.215 (0.536)	0.819 (0.107)	0.939 (-0.036)	1.000 (0.000)
Richea continentis	0.471 (0.299)	0.243 (-0.467)	0.056 (-0.695)	0.183 (0.5245)	0.352 (-0.381)	0.037 (-0.738)	0.194 (0.512)	0.729 (-0.146)	0.108 (-0.610)	0.207 (-0.500)	0.911 (-0.048)	0.289 (0.429)
Tasmannia xerophila	--	--	--	--	--	--	--	--	--	--	--	--

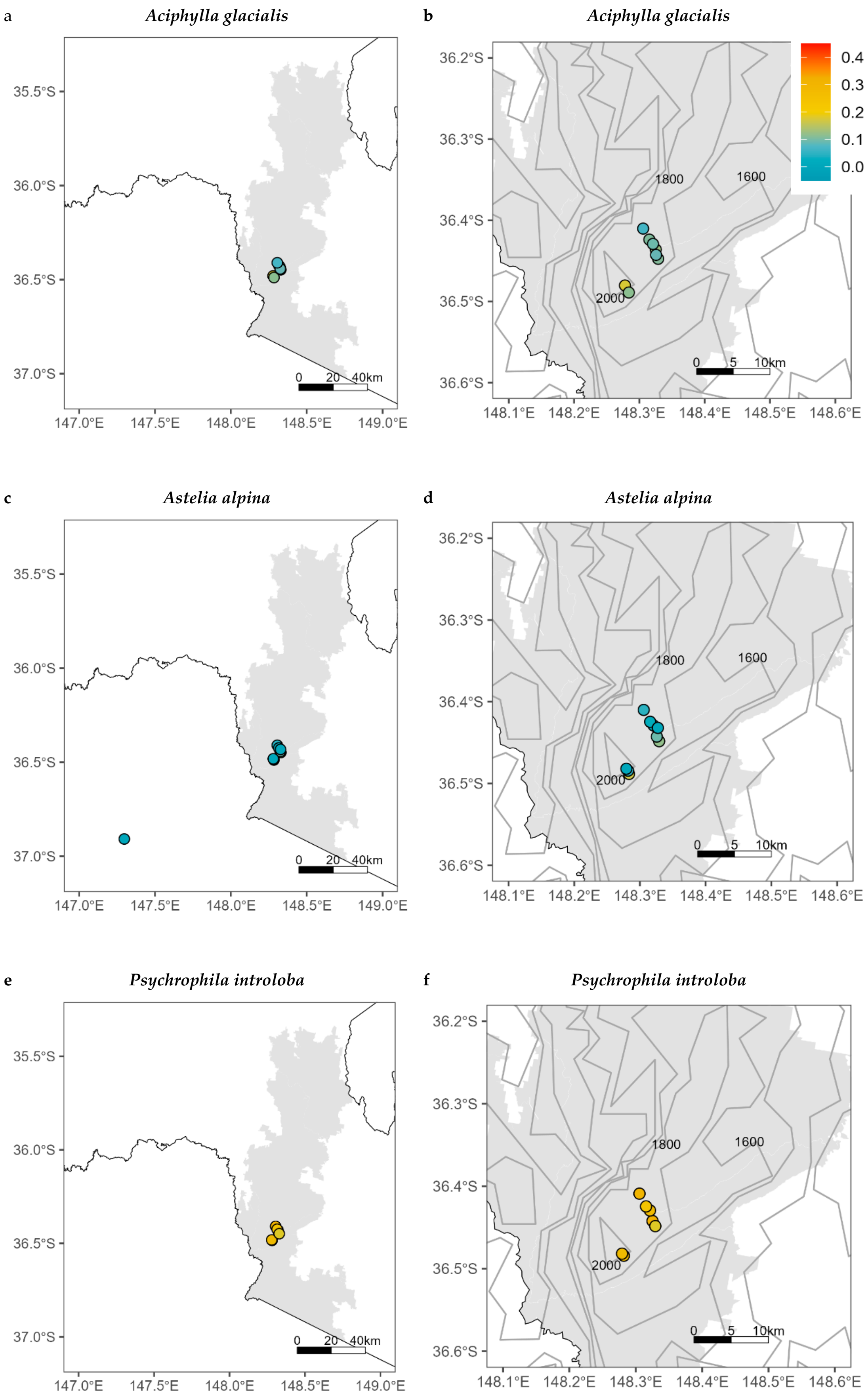

a
Aciphylla glacialis
b
Aciphylla glacialis
0.4
0.3
0.2
0.1
0.0
1800
1600
2000
c
Astelia alpina
d
Astelia alpina
e
Psychrophila introloba
f
Psychrophila introloba
35.5°S
36.0°S
36.5°S
37.0°S
147.0°E
147.5°E
148.0°E
148.5°E
149.0°E
0 20 40km
36.2°S
36.3°S
36.4°S
36.5°S
36.6°S
148.1°E
148.2°E
148.3°E
148.4°E
148.5°E
148.6°E
0 5 10km

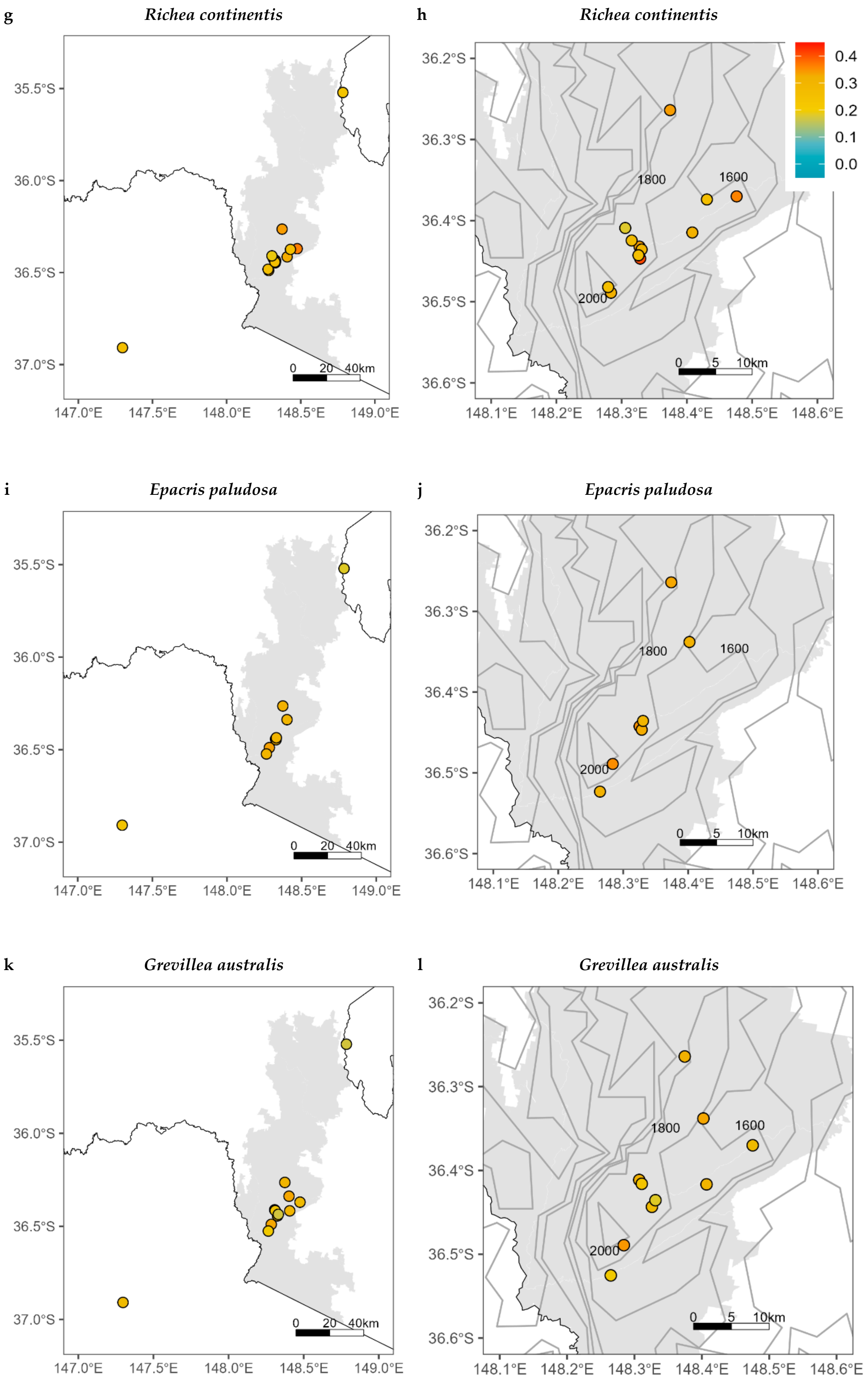

g
Richea continentis
35.5°S
36.0°S
36.5°S
37.0°S
0 20 40km
147.0°E 147.5°E 148.0°E 148.5°E 149.0°E
h
Richea continentis
36.2°S
36.3°S
36.4°S
36.5°S
36.6°S
1800
1600
2000
0 5 10km
148.1°E 148.2°E 148.3°E 148.4°E 148.5°E 148.6°E
0.4
0.3
0.2
0.1
0.0
i
Epacris paludosa
35.5°S
36.0°S
36.5°S
37.0°S
0 20 40km
147.0°E 147.5°E 148.0°E 148.5°E 149.0°E
j
Epacris paludosa
36.2°S
36.3°S
36.4°S
36.5°S
36.6°S
1800
1600
2000
0 5 10km
148.1°E 148.2°E 148.3°E 148.4°E 148.5°E 148.6°E
k
Grevillea australis
35.5°S
36.0°S
36.5°S
37.0°S
0 20 40km
147.0°E 147.5°E 148.0°E 148.5°E 149.0°E
l
Grevillea australis
36.2°S
36.3°S
36.4°S
36.5°S
36.6°S
1800
1600
2000
0 5 10km
148.1°E 148.2°E 148.3°E 148.4°E 148.5°E 148.6°E

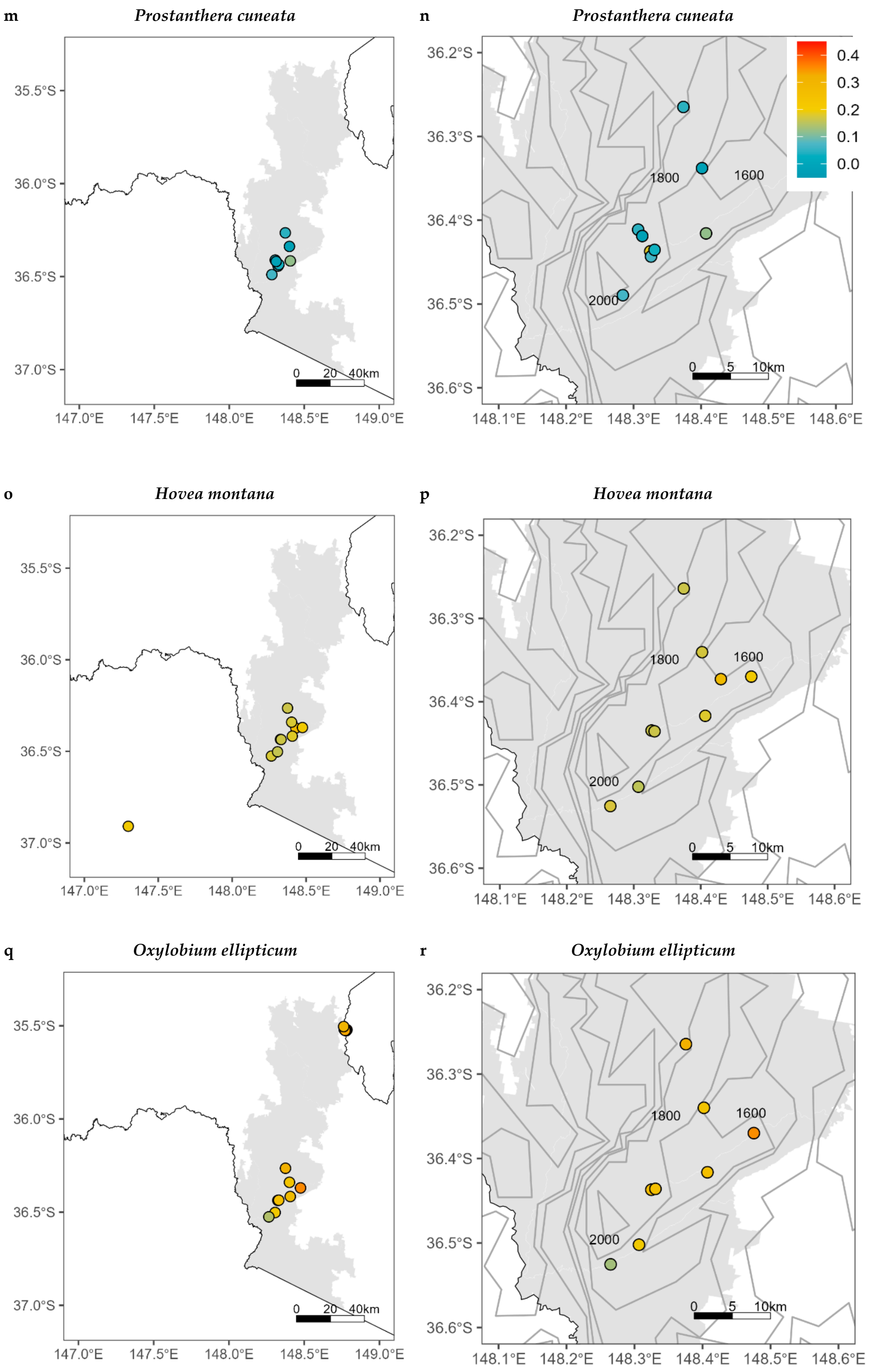

m
Prostanthera cuneata
n
Prostanthera cuneata
o
Hovea montana
p
Hovea montana
q
Oxylobium ellipticum
r
Oxylobium ellipticum
35.5°S
36.0°S
36.5°S
37.0°S
147.0°E
147.5°E
148.0°E
148.5°E
149.0°E
0 20 40km
36.2°S
36.3°S
36.4°S
36.5°S
36.6°S
148.1°E
148.2°E
148.3°E
148.4°E
148.5°E
148.6°E
0 5 10km
1800
1600
2000
0.4
0.3
0.2
0.1
0.0

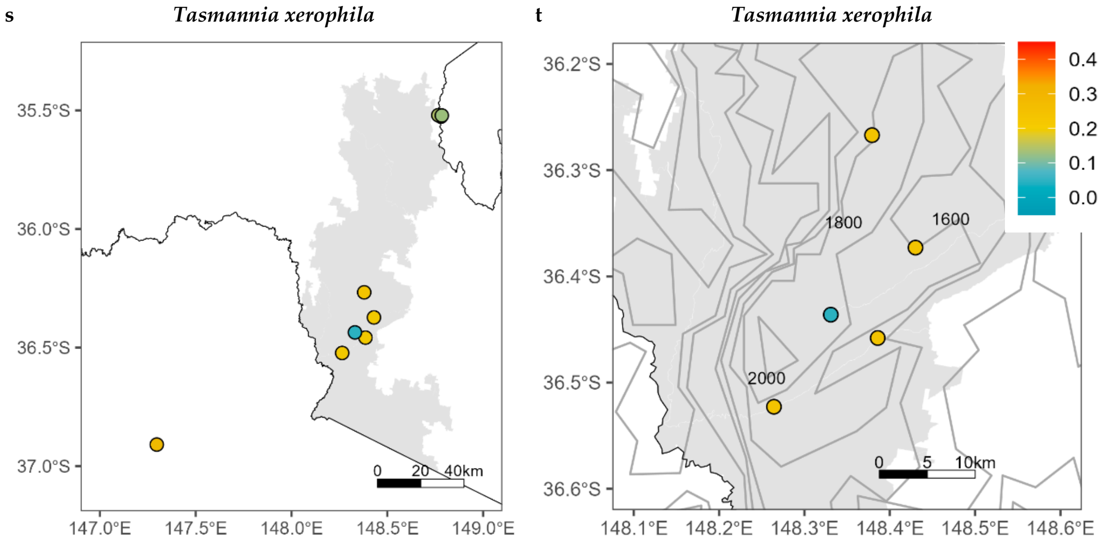

Figure S4. Within-population inbreeding (F_{is}) of alpine plants across south-eastern Australia (left panels) and within Kosciuszko National Park, NSW (right panels). Colours of circles indicates increasing F_{is}. The grey area represents the boundary of Kosciuszko National Park and the black borders represent state boundaries for ACT, NSW and VIC. On the right panels, the grey contour lines represent elevation with intervals of 200m. The study regions are Namadgi, ACT, Kosciuszko National Park, NSW, and Alpine National Park, Victoria.

Table S10. Average evaluation metrics and their standard deviation (in paranthesis), indicating the overall performance of the general linear model (GLM) and random forest (RF) models for three focal species: *Astelia alpina*, *Richea continentis* and *Epacris paludosa.* Each model algorithm underwent 50 runs (100 runs total).

**Species**	**Algorithm**	**Model validation**		**Sensitivity**	**Specificity**	**Percentage of models kept (over 50 runs)**
Astelia alpina	GLM	ROC	0.877 (0.03)	86.122 (3.61)	81.988 (3.28)	
		TSS	0.604 (0.07)	86.122 (3.79)	81.697 (3.67)	50%
	RF	ROC	0.922 (0.03)	99.927 (0.29)	99.103 (0.65)	
		TSS	0.688 (0.09)	99.902 (0.33)	99.055 (0.66)	84%
Richea continentis	GLM	ROC	0.979 (0.01)	97.460 (0.68)	96.120 (0.76)	
		TSS	0.911 (0.03)	97.487 (0.75)	96.053 (0.84)	100%
	RF	ROC	0.979 (0.01)	99.539 (0.82)	98.580 (0.77)	
		TSS	0.909 (0.04)	99.500 (0.87)	98.547 (0.82)	100%
Epacris paludosa	GLM	ROC	0.963 (0.006)	90.885 (0.85)	90.505 (0.67)	
		TSS	0.805 (0.02)	90.821 (0.86)	90.458 (0.67)	100%
	RF	ROC	0.987 (0.003)	99.867 (0.13)	99.623 (0.15)	
		TSS	0.878 (0.02)	99.860 (0.14)	99.603 (0.13)	100%
